# Tandem dye-sensitized solar cells achieve 12.89% efficiency using novel organic sensitizers

**DOI:** 10.1038/s41598-024-75959-0

**Published:** 2024-10-30

**Authors:** Safa A. Badawy, Ehab Abdel-Latif, Mohamed R. Elmorsy

**Affiliations:** 1 Department of Chemistry, Faculty of Science, Mansoura University, Mansoura, 35516 Egypt; 2 Department of Chemistry, Faculty of Science, New Mansoura University, New Mansoura, 35712 Egypt

**Keywords:** Tandem dye-sensitized solar cells (T-DSSCs), Triazatruxene (TAT), N719, Power conversion efficiency (PCE), Chemistry, Materials science

## Abstract

This study presents a significant advancement in tandem dye-sensitized solar cells (T-DSSCs) through the strategic synthesis of novel triazatruxene (TAT) sensitizers **MS-1** and **MS-2**. These organic sensitizers demonstrate exceptional light-harvesting capacity and overall performance, pushing the boundaries of power conversion efficiency (PCE) in DSSCs. The **MS-1**-based DSSCs achieved an impressive PCE of 12.81%, while **MS-2** sensitizers reached a notable 10.92%. These efficiencies represent significant improvements over the conventional **N719** dye (7.60%), demonstrating the potential of metal-free organic sensitizers in DSSC technology. The key to these noteworthy results lies in the molecular design of the organic sensitizers. The triazatruxene donor segment in the **MS-1** and **MS-2** dyes, featuring a rigid structure and efficient intramolecular charge transfer (ICT), proved to be a game-changer for photovoltaic properties. Building on these results, we explored an innovative parallel tandem cell (PT-DSSC) configuration. By connecting separate cells containing **N719** and **MS-1** sensitizers, we achieved a record efficiency of 12.89% with enhanced short-circuit current density (*J*_*SC*_) and open-circuit voltage (*V*_*OC*_)compared to single-dye cells. This study highlights the potential of molecular engineering in organic sensitizers and device optimization to enhance DSSC performance, paving the way for further advancements in solar cell technology.

**Supplementary Information:**

The online version contains supplementary material available at 10.1038/s41598-024-75959-0.

## Introduction

**Figure 1:**
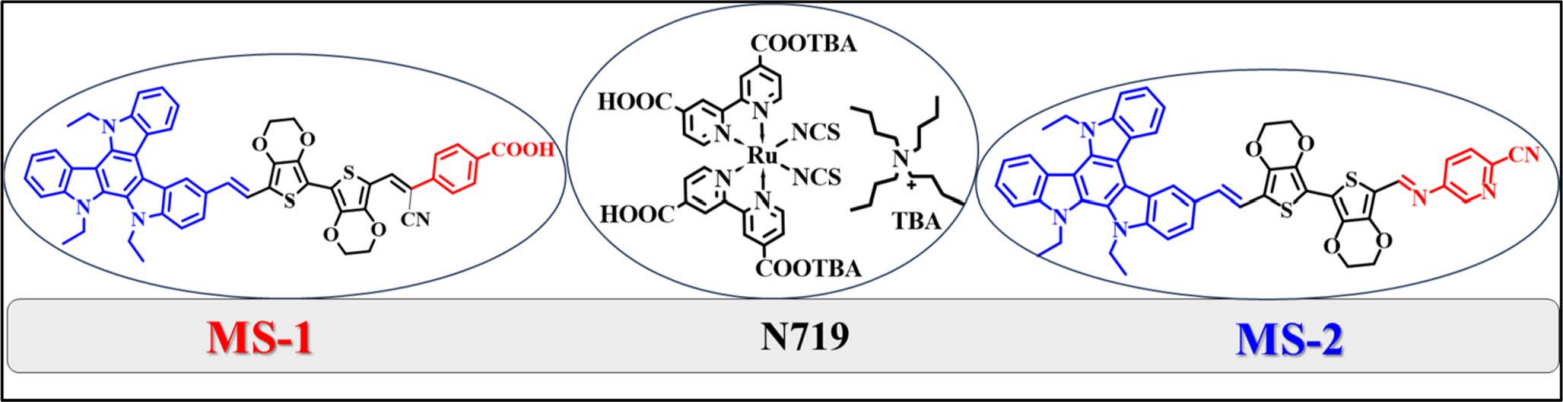
Chemical structures of sensitizers **MS-1-2** and **N719.**

Over the past few decades, dye-sensitized solar cells (DSSCs) have attracted significant attention as promising photovoltaic systems for renewable energy generation. This is because of their unique characteristics, such as cost-effectiveness, flexibility, and ease of manufacture^1–3^. Since their discovery in 1991, efficient photosensitizers have been continuously developed, opening numerous possibilities for their design. Photosensitizers are crucial components in DSSCs because they play a vital role in determining the performance of photovoltaic devices^4^. Various strategies have been employed to improve the power conversion efficiency (PCE) of DSSCs, such as broadening the absorption spectra and increasing the molar extinction coefficient to capture more photons from the solar radiation. However, the limitations of effective electron injection at the dye/nanoparticle interface and energy losses, particularly during the electron injection process, have hindered the overall performance of DSSCs. Organic dyes, with their diverse molecular designs, abundant raw materials, and vibrant colors, offer solutions to these issues through appropriate molecular engineering^5^. The choice of the π-segment in dyes plays a crucial role in determining not only their electronic properties but also the energetic and kinetic attributes of the titania/dye/electrolyte interface during the electron injection and regeneration processes. This process ultimately determines the performance of a solar cell. However, electron injection from an excited dye into TiO_2_ is a complex process and a major source of energy loss in DSSCs. This process involves dye excitation, the vibrational relaxation of the excited dye, and electron injection. Previous studies have indicated that sensitizers with higher flexibility and more double bonds tend to exhibit lower power conversion efficiency (PCE)^6^. This is because the electron injection process competes with vibrational relaxation. Parallel Tandem dye-sensitized solar cells (PT-DSSCs) are often used to enhance the sunlight-harvesting capacity of solar cells. These cells consist of two separate DSSCs connected in a series or parallel arrangement, with the top and bottom cells operating in tandem^7^. PT-DSSCs have a unique structure that eliminates the need to mix multiple dyes during co-sensitization. This structure overcomes issues related to competitive adsorption and difficulties in achieving the appropriate concentration ratio, timing, and sequence of dyes commonly encountered in co-sensitization^8^. PT-DSSCs have demonstrated noteworthy performance metrics, particularly high power conversion efficiency (PCE), which is largely attributed to the broader absorption spectrum interval resulting from increased light absorption between the top and bottom cells^9^. Moreover, studies have demonstrated that PT-DSSCs exhibit superior performance in enhancing the efficiency compared to series tandem DSSCs (ST-DSSCs)^10^. According to Kim et al., a noteworthy power conversion efficiency (PCE) of 14.64% was attained in PT-DSSCs employing the organic dye SGT-137 and zinc porphyrin dye SGT-021^11^. The widely used ruthenium dye **N719** has garnered significant attention owing to its exceptional photostability and high power conversion efficiency (PCE)^12^. However, the relatively low molar extinction coefficient (ε) of N719 may hinder its further development^13^. The geometry of the sensitizer molecule is critical for efficient charge injection and minimization of vibrational relaxation, which can result in energy loss in DSSCs. The rigid TAT structure enhanced the DSSC efficiency, achieving a PCE of 13.6%, which is the highest reported for a single-sensitizer DSSC. These results underscore the importance of molecular design in optimizing the DSSC performance^14^. Although triazatruxene (TAT) has been widely used in various applications, including OLEDs and perovskite solar cells, its exceptional properties, such as high molar absorption coefficient, excellent hole transport capability, and photostability, have not resulted in many highly efficient photosensitizers for liquid and solid-state DSSCs. We synthesized and characterized two distinct TAT-based sensitizers (MS-1 and **MS-2**) as shown in (Fig. 1). These dyes were designed by incorporating triazatruxene as a donor segment linked to 3,4-ethylenedioxythiophene (EDOT) and a carboxyphenylacetonitrile (-CAN) acceptor for **MS-1** and 5-aminopicolinonitrile (-APN) for **MS-2**. Furthermore, the UV-vis absorption spectrum demonstrated cooperative absorption between **MS-1**,** MS-2**, and N719, suggesting that these dyes are ideal candidates for PT-DSSCs. Based on these advantages, we evaluated the performance of PT-DSSCs based on **MS-1 + N719** sensitizers and compared them with those of DSSCs based on **MS-1**,** MS-2**, and **N719.** The results showed that PT-DSSCs based on **MS-1 + N719** sensitizers achieved higher PCEs (13.60%) than those based on **MS-1** (12.8%), **MS-2** (10.92%), and **N719** (7.60%), making them promising candidates for DSSCS applications.

### Experimental section

Comprehensive details, including photovoltaic measurements, synthetic procedures for all structures, and extensive structural characterization of all fabricated devices, are provided in the Supporting Information (Figures S1-S26). This supplementary material offers a thorough foundation for our findings and ensures the reproducibility of our work.

## Results and discussion

### Chemistry

**Figure 2:**
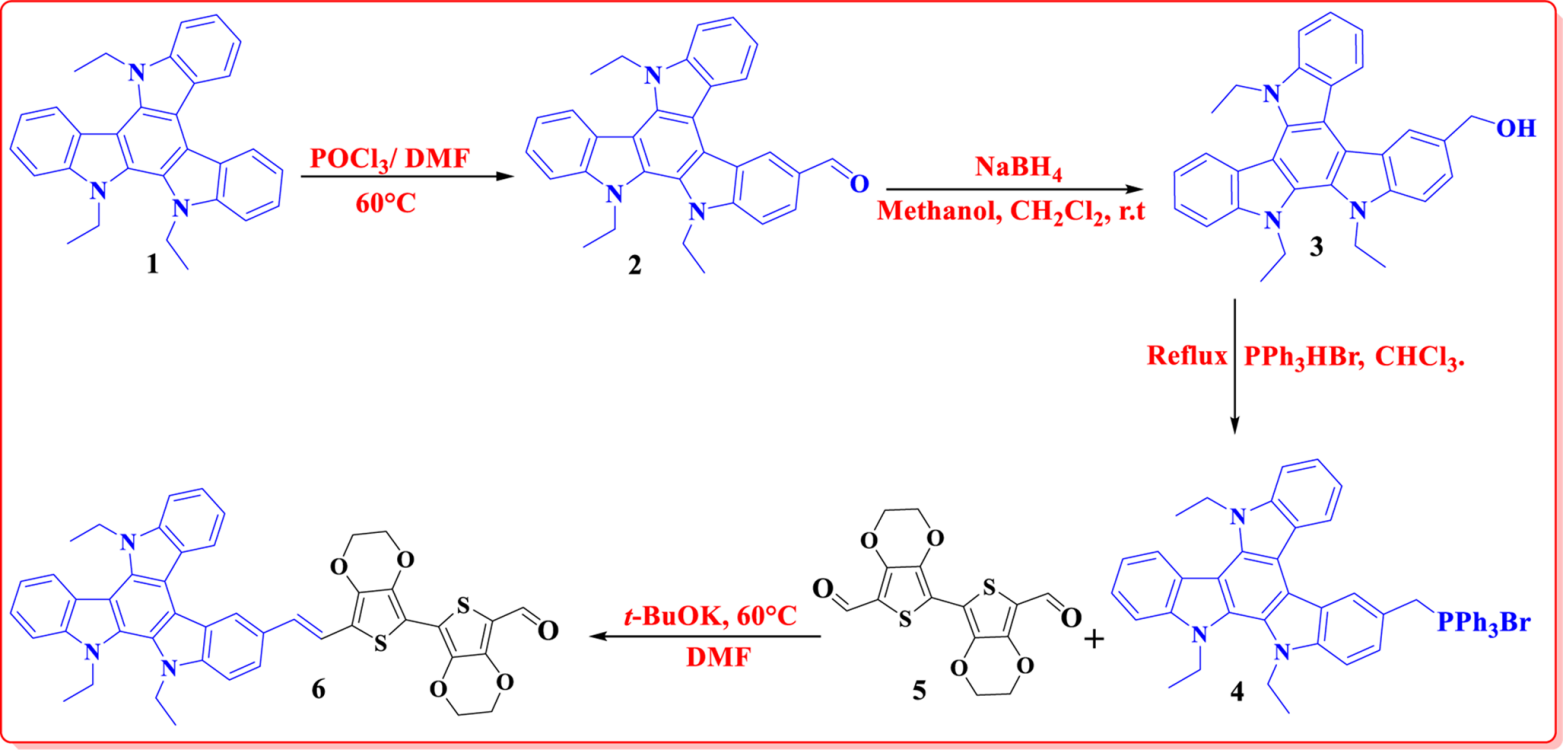
Synthesis of triazatruxene-3,4-ethylenedioxythiophene aldehyde (**6)**.

**Figure 3:**
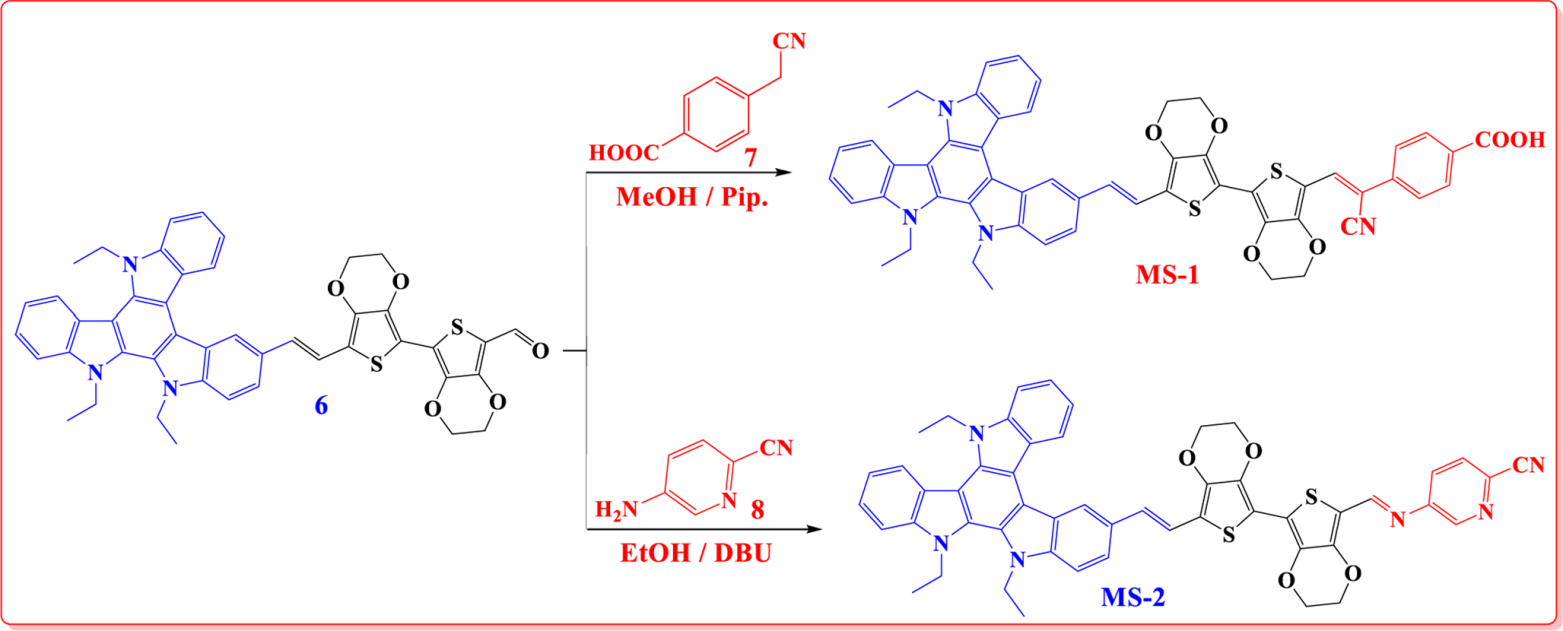
Synthetic route to **MS-1-2** sensitizers.

The synthetic routes to the novel **TAT-**based organic sensitizers (**MS-1-2**) are illustrated in Figs. 2 and 3. Figure 2 shows the synthetic pathway for TAT-aldehyde **(6).** Initially, the triazatruxene core **(1)** was synthesized through intermolecular condensation of 2-indolinone, which is commercially accessible. This process was performed in a single step to produce a planar aromatic core. Subsequently, n-ethyl chains were attached to the core, as documented in a previous study^15^. Following the Vilsmeier-Hack reaction, the triazatruxene core was formylated, resulting in the production of TAT-aldehyde, as shown in Fig. 2. TAT-aldehyde **(2)** was reduced to its corresponding reduced alcohol compound **(3)** in a mixed solvent system of methanol and dichloromethane in the presence of NaBH_4_. Building upon the successful synthesis of compound **3**, novel phosphonium salt **4** was prepared through a reaction with triphenylphosphine hydrobromide in chloroform. Then, a wittig reaction occurred between the salt of compound (**4)** and 2,2’,3,3’-tetrahydro-[5,5’-bithieno[3,4-b][1,4]dioxine]-7,7’-dicarbaldehyde **(5)**, which was prepared as previously described^16^ to synthesize TAT-aldehyde **(6).** All the complete synthetic data for the compounds are presented in the supplementary file.

In accordance with Fig. 3, sensitizers **MS-1-2** were synthesized through Schiff base reactions involving **TAT-**aldehyde **(6)**, 4-(cyanomethyl)benzoic acid **(7) in** methanol, drop base catalyst piperidine, and refluxing in ethanol and DBU with 5-aminopicolinonitrile **(8).** Subsequently, recrystallization was performed to purify the resulting dye sensitizers and intermediates. The molecular structures of the synthesized dyes (**MS-1-2**) and their intermediates were characterized using various spectroscopic techniques. The IR spectra of **MS-1** showed characteristic absorption bands at 2995, 2920 for aliphatic groups (C-H), 2207 (-CN), 1687 (C = O) and 1619 cm⁻¹ (C = C), confirming the presence of the cyano group and carbonyl bond of carboxylic acid group, respectively, Furthermore, ^1^HNMR spectra showed aliphatic protons of ethyl group of triazatruxene (TAT) moieties as muliplet signals from 1.36 to 1.39 for (3CH_3_) and 4.30–4.36 for (3CH_2_) ppm, for the protons of EDOT moiety appeared from 4.21 to 4.24 ppm. Additionally, a characterized doublet signal at δ 7.03 ppm was linked to the two vinylic protons (*J* = 12.00 Hz), and olefinic proton appearing as a singlet at δ 7.54 ppm (= CH). Furthermore,^13^CNMR showed a characteristic signal for aliphatic group at 13.33, 43.92, 57.01, 66.72 ppm of the carbons of ethyl groups and EDOT ring. Additionally, signal at 118.16 ppm for cyano group (CN) and 168.95 ppm for the carbon of carbonyl group of (COOH). Similarly, a novel sensitizer (**MS-2**) was successfully synthesized through a condensation reaction between TAT-aldehyde **(6)** and 5-aminopicolinonitrile. The reaction was carried out under reflux in ethanol for 28 h using (DBU) as a basic catalyst. The structure of **MS-2** was confirmed by various spectroscopic techniques. The melting point of the compound was found to–224–226 °C. The IR spectrum showed characteristic peaks at 2924 and 2841 cm⁻¹, corresponding to C-H stretching vibrations. The presence of a nitrile group was confirmed by the sharp peak at 2211 cm⁻¹, while the peak at 1620 cm⁻¹ was attributed to C = C stretching. ¹H NMR spectroscopy provided further evidence for the successful synthesis. The presence of ethyl groups was confirmed by the multiplet at 1.36–1.39 ppm for (9 H) of three methyl groups of TAT, the signals at 4.21–4.24 ppm and 4.30–4.36 ppm (14 H total) for the methylene protons for the ethyl group and EDOT moiety. The characterized singlet signal for the protons of the vinylic group at 7.05 *J* = 12.00 Hz. The pyridine ring exhibits two distinct signals, a doublet signal at 8.45 and 8.74 ppm, coupled with a coupling constant (*J*) of 4.00 Hz. In addition, a singlet signal from the pyridine ring appears at 8.74 ppm. The imine proton (CH = N) appeared as a singlet at 8.66 ppm, confirming the formation of a Schiff base. ¹³CNMR spectroscopy further corroborated the structure, showing the expected number of carbon signals. The spectrum revealed peaks for aliphatic carbons at 14.34, 44.94, 54.72, and 67.73 ppm, and the signal at 117.58 ppm can be attributed to the nitrile carbon. For imine carbon (CH = N) appear at 150.10 ppm.

### Photophysical properties

**Figure 4:**
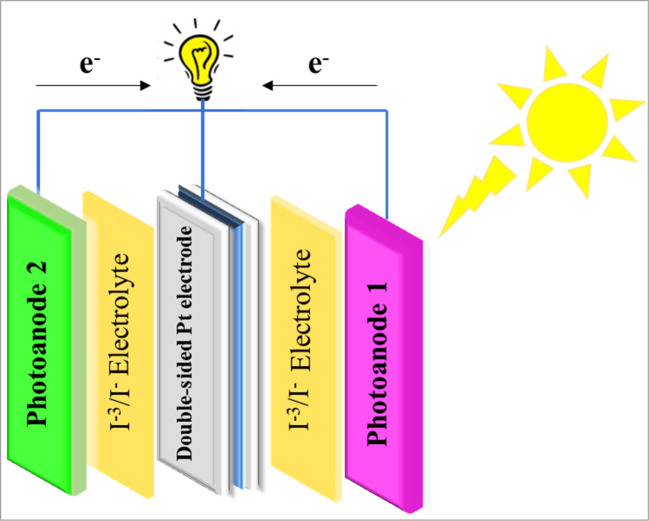
Device structure of PT-DSSCs with double-sided transparent Pt electrodes.

To verify our hypothesis and assess the applicability of **MS-1-2** in DSSCs, a series of photophysical and electrochemical tests were performed. Additionally, an unconventional PT-DSSC featuring a double-sided transparent Pt electrode was constructed and investigated in this study (Fig. 4). Furthermore, the innovative design of PT-DSSCs enabled more effective harvesting of incident light, thereby enhancing the overall efficiency of the device.

Overall, the device structure of a PT-DSSC with double-sided transparent Pt electrodes involves a tandem configuration of two cells, each optimized for a specific wavelength range. The transparent Pt electrodes on both sides of the device enable efficient electron collection and facilitate current flow in the solar cell^17^. Subsequently, **N719** and **MS-1** were employed as sensitizers in double-sided PT-DSSCs, and their placement order was varied to investigate their impact on device performance.

**Figure 5:**
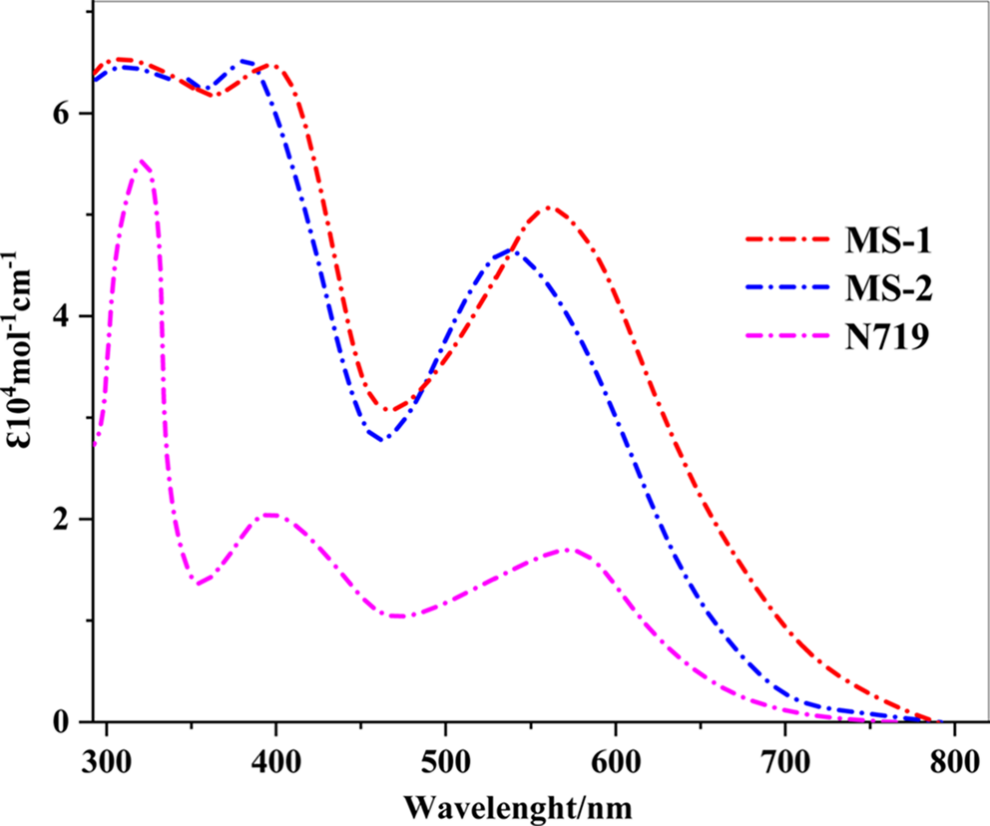
UV-Vis absorption spectra of **MS-1-2 and N719** sensitizers in DMF solution.

The absorption spectra of the two synthesized dyes, **MS-1 and MS-2**, in dimethylformamide (DMF) solution with a concentration of 2 × 10^−5^ M are shown in Fig. 5. The key performance metrics and corresponding data are summarized in Table 1, which offers a clear overview of our experimental results. Sensitizer **MS-1** contains a triazatruxene (TAT) donor segment with a larger coplanar π-system structure, which could potentially enhance electron donation across the sensitizer skeleton.

**Table 1:** UV-Vis absorption profiles of TAT-sensitizer **MS-1-2**.

Sensitizers	λ_max_ (nm)	Ɛ (10^4^ M^−1^cm^−1^)	λ_onset_ / nm	Experimental E_0−0_ (eV)
**MS-1**	**396**,** 556**	**6.61**,** 5.23**	**739**	**1.67**
**MS-2**	**385**,** 544**	**6.60**,** 4.92**	**676**	**1.83**

Both sensitizers **(MS-1-2)** showed two distinct absorption bands^18^. The substantial absorption in the visible spectrum (450–600 nm) is ascribed to intramolecular charge transfer (ICT) between the highly donating triazatruxene-3,4-ethylenedioxythiophene and acceptor groups, namely carboxyphenylacetonitrile (-CAN) for **MS-1** and 5-aminopicolinonitrile (-APN) for **MS-2.** Interestingly, the absorption maxima (λ_max_) of the D-π-A sensitizers were arranged in the following sequence: **MS-1** (556 nm, ε = 5.23 × 10^4^ M^−1^ cm^−1^) > **MS-2** (544 nm, ε = 4.92 × 10^4^ M^−1^ cm^−1^). The molar extinction coefficients (ε) of **MS-1** and **MS-2** demonstrated a substantial improvement compared to that of the benchmark dye **N719**, suggesting a noteworthy capacity for light absorption, as shown in Fig. 5^19^. The significant absorption of these dyes is primarily attributed to the triazatruxene moiety attached to the appropriate functional groups in **MS-1** and **MS-2**. Modification of the anchoring moiety from carboxyphenylacetonitrile (-CAN) to 5-aminopicolinonitrile (-APN) resulted in a slight and consistent shift of the intramolecular charge transfer (ICT) transition peaks. Specifically, the peak wavelength decreases from 556 nm for **MS-1** to 544 nm for **MS-2.** This spectral shift can be attributed to the difference in the electron-withdrawing ability of the anchoring groups, with -CAN exhibiting a stronger electron-withdrawing effect than that of-APN. This broadening of the absorption band is often associated with the improved light-harvesting efficiency of the sensitizer, as it allows the sensitizer to absorb a wider range of incident light, resulting in a higher probability of excitation and enhanced light absorption.

**Figure 6:**
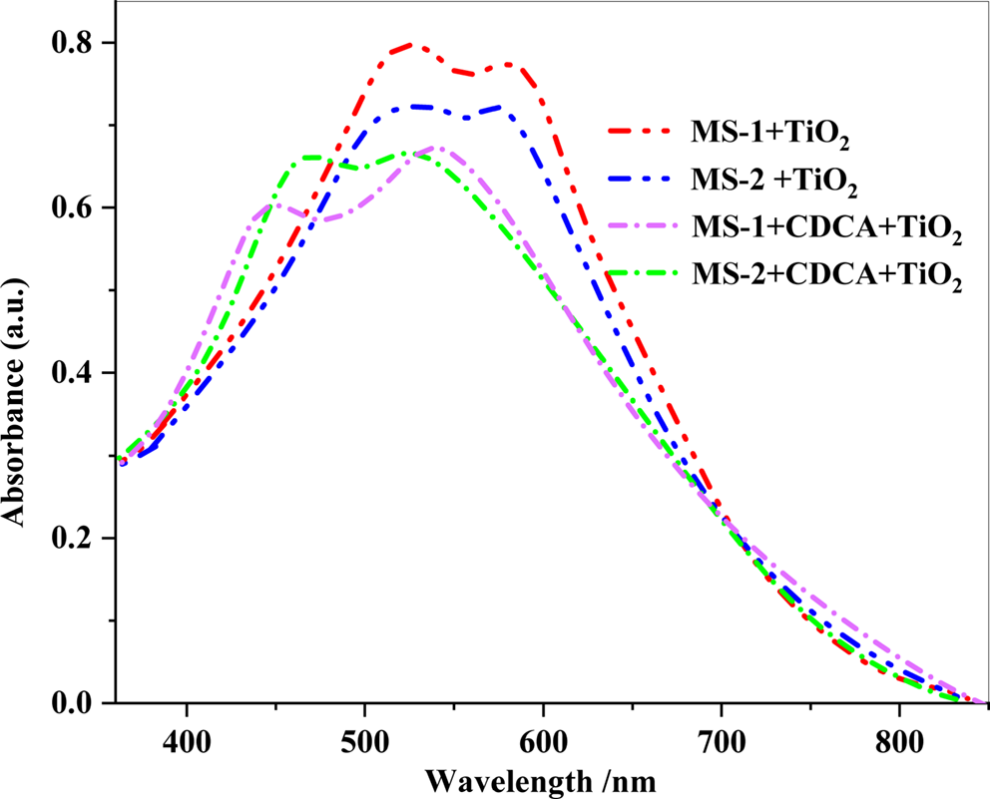
UV-visible spectra of **MS-1-2** and **MS-1-2** with CDCA on TiO_2_.

After evaluating the absorption spectra of the dyes, it became apparent that **MS-1-2** demonstrated a wider spectrum in the thin film when compared to its absorption spectra in the DMF solution. Additionally, the **MS-1-2** sensitizers exhibited a bathochromic shift in their absorption maximum upon adsorption on the nanocrystalline TiO_2_ film, as observed in the range of (350–600 nm) in Fig. 6^15^. The probable cause of the spectral bathochromic shift of the dyes attached to the TiO*2* film is J-type aggregation, which is advantageous for enhancing the light harvesting efficiency^20^. *J*-type aggregation of **MS-1-2** was created by the introduction of strong electron-withdrawing groups. The strong interaction of the anchoring groups of **MS-1-2** with the surface of the titanium ion enhances the conjugation and results in a decrease in the energy level of the π*-orbital of sensitizers. These characteristics enhance the light-harvesting capacity of dyes, resulting in higher PCEs of the (TAT) sensitizers^20^. Furthermore, it is anticipated that the increased dye loading on the TiO_2_ surface and the broader spectral coverage of **MS-1** sensitizers in the visible region compared to **MS-2** will result in improved light-harvesting capabilities and bolster the photocurrent in devices. Chenodeoxycholic acid (CDCA) was employed as a co-adsorbent to investigate its effects on dye aggregation and spectral properties. No significant difference was observed in the overall absorption intensity of TiO_2_ with and without CDCA. However, a notable blue shift in the absorption spectrum was detected when comparing (**MS-1-2 + CDCA**) to **MS-1-2** alone on TiO_2_. This spectral shift can be primarily attributed to the reduction in dye aggregation upon addition of CDCA. CDCA is known to prevent aggregation, which generally improves electron transfer between the dye and TiO_2_. This is supported by the observed blue shift in the UV spectra (Fig. 6), indicating a more efficient electronic coupling between the sensitizers and TiO_2_^21^.

### Theoretical calculations

**Figure 7:**
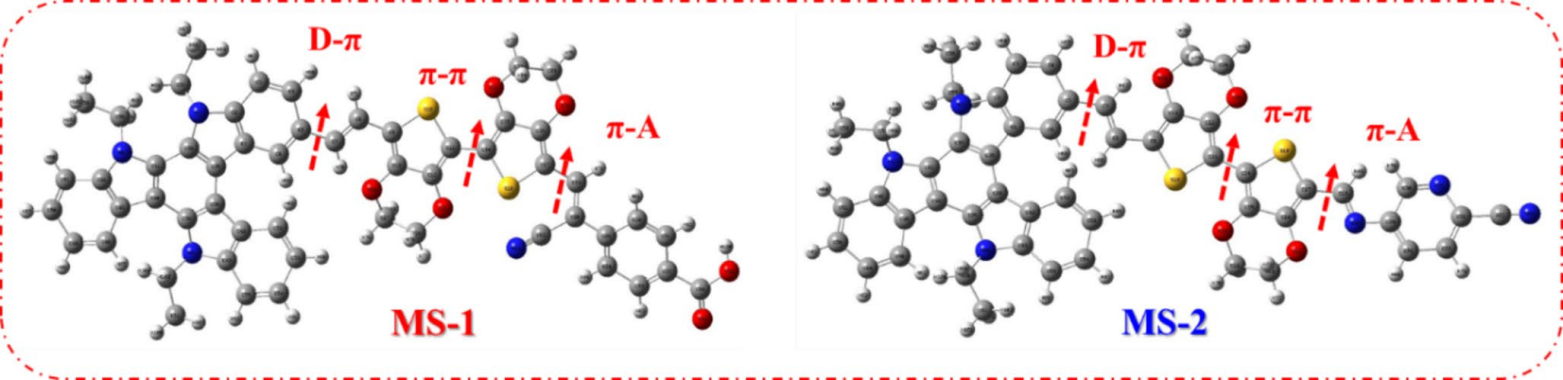
Optimized geometrical structures of sensitizers **MS-1-2**.

The optimal configurations of the dyes were determined at the B3LYP/6-31G(d, p) level of theory, as illustrated in Fig. 7. A comprehensive list of geometric parameters, including the dihedral angles and bond lengths, is presented in Table 2. As depicted in Fig. 7, sensitizers feature a transition from the donor (-TAT) on the left to the accepting group on the right, specifically carboxyphenylacetonitrile (-CAN) in **MS-1** and 5-aminopicolinonitrile (-APN) in **MS-2**, culminating in a segment connected to the 3,4-ethylenedioxythiophene linker. The dihedral angles for **MS-1-2** and **D–π** were slightly twisted in the range of 28.35°–37.69^°^ and for **π-A** 179^°^-179.27^°^, indicating the presence of steric hindrance between the donor and internal units (EDOT), which would be helpful in suppressing dye aggregation in the device and enhancing the thermal stability^22–24^. The research found that sensitizers demonstrated improved coplanarity between the π-spacer (EDOT) and the acceptor groups (CAN and APN).

**Table 2:** Dihedral angles and bond lengths of sensitizer **MS-1-2.**

Sensitizers	Dihedral angle (°)			Bond length (Å)		
	D–π (°)	π– π (°)	π-A (°)	D–π (Å)	π– π (Å)	π –A (Å)
**MS-1**	28.35	179.30	179.27	1.44	1.43	1.32
**MS-2**	37.69	176	179	1.75	1.46	1.63

The results indicate that the bond lengths of all sensitizers **MS-1-2** fall within the range of 1.43–1.75 Å. Å), and π-A bond lengths, **MS-1** as D-π (1.44 Å) and π-A (1.32 Å), and **MS-2** as D-π (1.75 Å) and π-A (1.63 Å). This arrangement facilitated efficient intramolecular charge transfer from the donor (TAT) to the anchoring groups. These findings imply that modifying the acceptor units in the sensitizer moieties can significantly enhance the planarity of the dyes under investigation, which is essential for their absorption and intramolecular charge transfer (ICT) properties.

#### Elucidating the determinants of theoretical properties in MS-1-2 molecular frameworks

To develop a comprehensive understanding of the relationship between the structure and activity of the recently synthesized dye **MS-1-2** as a photosensitizer, various chemical descriptors were calculated using Eqs. (1–8)^25^. Firstly, the ionization potential $\:\left(IP\right)\:$of the dye sensitizers plays a crucial role in determining the efficiency of electron injection from the excited dye into the conduction band of the TiO_2_ in the DSSC. A lower$\:\:IP\:$facilitates efficient electron transfer, as it reduces the energy barrier for electron injection., while the electron affinity $\:\left(EA\right)\:$affects its capacity to receive electrons^25^. As shown in Table 3, the sensitizer **MS-1** exhibited a lower *IP)* value than **MS-2** reached to 5.45 eV. This finding confirms that **MS-1** enhances the efficiency of (DSSCs)^25^. The chemical potential (µ) is a crucial factor in the electron transfer processes within DSSCs. It determines the driving force for electron movement from the dye molecules to the semiconductor material and through an external circuit. For the sensitizers MS-1-2, the chemical potential values range from − 4.49 to -4.55 eV. The chemical hardness (η) is related to the energy difference between (HOMO) and (LUMO) of the sensitizer. This represents the amount of energy required to either remove an electron from the HOMO or add an electron to the LUMO^26^. As shown (*η*) for the sensitizer follows the order **MS-2** (0.92 eV) > **MS-1** (0.86 eV). The chemical hardness showed that MS-1 had the lowest energy for electron movement. Therefore, the selection of the sensitizer molecule and its chemical hardness play crucial roles in optimizing the performance of (DSSCs). For the dyes studied, the values of softness (S) followed the order: **MS-1** (1.16 eV) > **MS-2** (1.08 eV)^27^. Sensitizers with higher electrophilicity (ω) tend to have a greater affinity for electrons, facilitating the transfer of electrons from the excited dye molecule to the conduction band of the semiconductor, where they contribute to the generation of electrical current. The electrophilicity values of (**MS-1-2**) were **as follows: MS-1** (11.91 eV) > **MS-2** (11.21 eV). The photovoltaic performance was affected by the electro-accepting power (ω+) and electron-donating power (ω^−^); the highest ω^+^ value indicates the highest electron-accepting ability. Table 3 shows that the order of increasing ω^+^ for the original and designed dyes was **MS-1 > MS-2**. The **MS-1** sensitizer exhibited the highest electron-accepting power and stabilization energy among the tested dyes. The study revealed a consistent pattern in the computed electron-donating capabilities (ω^−^) of the newly designed sensitizers **MS-1-2** ranging from **(13.60-14.26 eV)**, which aligned with their electrophilicity and electron-accepting characteristics. Among these, the sensitizer **MS-1** stood out for its superior performance. It exhibited the most favorable stabilization energy, demonstrated the highest efficiency in accepting electrons, and exhibited the lowest chemical hardness. These attributes contribute to an improvement in short-circuit current density(J_SC_) and ultimately lead to a higher power conversion efficiency (*PCE*) for **MS-1**^28^. Based on the previous chemical parameters of, it can be concluded that dyes incorporating the carboxyphenylacetonitrile (**-CAN**) moiety for **MS-1** exhibit superior performance in DSSCs.

1 $$\boldsymbol I\boldsymbol P\boldsymbol=\boldsymbol-{\boldsymbol E}_{\boldsymbol H\boldsymbol O\boldsymbol M\boldsymbol O}$$

2 $$\boldsymbol E\boldsymbol A\boldsymbol=\boldsymbol-{\boldsymbol E}_{\boldsymbol L\boldsymbol U\boldsymbol M\boldsymbol O}$$

3 $$\boldsymbol\eta\boldsymbol=\left(\frac{{\boldsymbol E}_{\mathit L\mathit U\mathit M\mathit O}\boldsymbol-{\boldsymbol E}_{\mathit H\mathit O\mathit M\mathit O}}{\mathbf2}\right)$$

4 $$\boldsymbol\chi\boldsymbol=\boldsymbol-\boldsymbol\mu\boldsymbol=\boldsymbol-\left({\boldsymbol E}_{\boldsymbol L\boldsymbol U\boldsymbol M\boldsymbol O}+{\boldsymbol E}_{\boldsymbol H\boldsymbol O\boldsymbol M\boldsymbol O}\right)\boldsymbol/\mathbf2$$

5 $$\boldsymbol S\boldsymbol=\left(\mathbf1\boldsymbol/\mathbf\eta\right)$$

6 $$\boldsymbol\omega\boldsymbol=\frac{\left(\boldsymbol I\boldsymbol P\boldsymbol+\boldsymbol E\boldsymbol A\right)^{\mathbf2}}{\mathbf4\left(\boldsymbol I\boldsymbol P\boldsymbol-\boldsymbol E\boldsymbol A\right)}$$

7 $$\boldsymbol\omega^+\boldsymbol=\frac{\left(\boldsymbol I\boldsymbol P+\mathbf3\boldsymbol E\boldsymbol A\right)^{\mathbf2}}{\mathbf{16}\left(\boldsymbol I\boldsymbol P-\boldsymbol E\boldsymbol A\right)}$$

8 $$\mathbf\omega^-\boldsymbol=\frac{\left(\boldsymbol3\boldsymbol I\boldsymbol P+\boldsymbol E\boldsymbol A\right)^{\mathbf2}}{\mathbf{16}\left(\boldsymbol I\boldsymbol P-\boldsymbol E\boldsymbol A\right)}$$

**Table 3:** Chemical reactivity descriptors of **MS-1-2** sensitizers.

Dye	HOMO (eV)	LUMO (eV)	E_0−0_ (eV)	IP (eV)	EA (eV)	η (eV)	χ (eV)	µ (eV)	S (eV)	ω (eV)	ω^+^ (eV)	ω^−^ (eV)
**MS-1**	**-5.35**	**-3.63**	**1.72**	**5.35**	**3.63**	**0.86**	**4.49**	**-4.49**	**1.16**	**11.91**	**9.76**	**14.26**
**MS-2**	**-5.49**	**-3.65**	**1.84**	**5.49**	**3.65**	**0.92**	**4.55**	**-4.55**	**1.08**	**11.21**	**9.05**	**13.60**

### Molecular modeling

**Figure 8:**
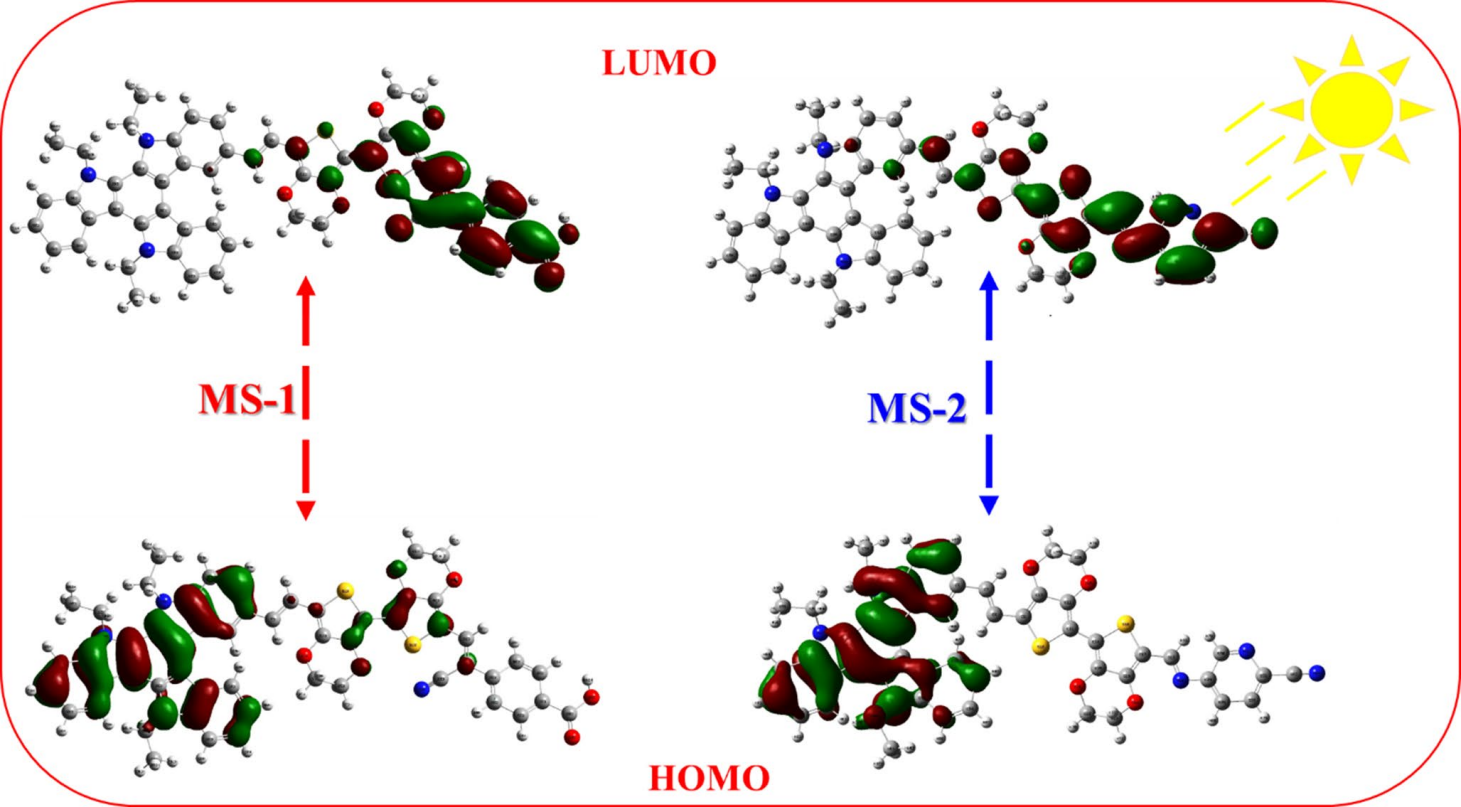
FMO distribution of the** MS-1-2** sensitizers.

The efficiency of intramolecular charge transfer (ICT) is shown in Fig. 8. In the HOMO configuration, the electron density was uniformly distributed along the strong electron-donating triazatruxene (**TAT**) core and 3,4-ethylenedioxythiophene (EDOT) π-bridging units. Conversely, the LUMO reveals localized electron distributions primarily on the acceptor moieties- carboxyphenylacetonitrile (-**CAN**) for **MS-1** and 5-aminopicolinonitrile (-**APN)** for **MS-2** and their adjacent π-spacers. This distribution pattern results from the intramolecular charge transfer along the π-conjugated skeleton. The observed electron distribution characteristics enabled both sensitizers **MS-1-2** to efficiently inject electrons into the conduction band of TiO_2_, demonstrating their potential effectiveness in dye-sensitized solar cell applications.

**Figure 9:**
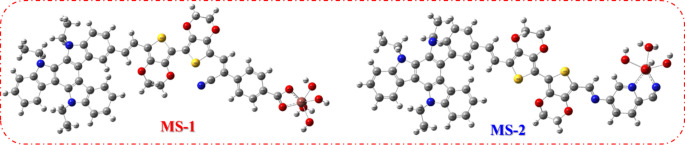
Interaction of sensitizers **MS-1-2** with Ti(OH)_3_H_2_O.

**MS-1-2** dyes exhibit intramolecular charge transfer (ICT) from various donor groups to acceptor moieties, facilitated by a π-spacer. This ICT process promotes spatial separation of the HOMO and LUMO energy levels upon light absorption, enhancing electron injection from the excited dye molecules to the TiO_2_ surface and resulting in improved photovoltaic performance. To model dye adsorption on TiO_2_, we utilized the Ti(OH)_3_H_2_O moiety^29^, Ti(OH)_3_H_2_O exhibits octahedral titanium atoms with two positions occupied by oxygen atoms. During the transition from the dye to the titanium complex, acidic hydrogen atoms migrate to the titanium complex. Figure 9 illustrates the fully optimized geometry of the dye@Ti(OH)_3_H_2_O complexes, providing insight into the molecular arrangements at the dye-TiO_2_ interface.

### Electrochemical properties

The electrochemical characteristics of the sensitizers were studied using cyclic voltammetry (CV) to examine the frontier orbital levels. The ground-state oxidation potential (GSOP) was determined directly from the CV curves shown in (Fig. S27) in the Supplementary File. The excited-state oxidation potential (ESOP) was then calculated using the optical bandgap (E_0−0_) obtained from the onset of the absorption spectra (Fig. 5).

The GSOP (equivalent to the HOMO level) was calculated using the equation:

9 $$\boldsymbol G\boldsymbol S\boldsymbol O\boldsymbol P=-\left(\boldsymbol E_{\boldsymbol o\boldsymbol n\boldsymbol e\boldsymbol s\boldsymbol t}^{\boldsymbol o\boldsymbol x\boldsymbol d}\boldsymbol+4.7\right)$$

Where $\:{\varvec{E}}_{\varvec{o}\varvec{n}\varvec{e}\varvec{s}\varvec{t}}^{\varvec{o}\varvec{x}\varvec{d}}$ is the onset oxidation potential vs. NHE.

The ESOP was then calculated using:

10 $$\boldsymbol E\boldsymbol S\boldsymbol O\boldsymbol P=\left(\boldsymbol G\boldsymbol S\boldsymbol O\boldsymbol P-{\boldsymbol E}_{\boldsymbol0\boldsymbol{\mathit-}\boldsymbol0}\right)$$

The LUMO levels were estimated by subtracting ***E***_***0–0***_ from the GSOP.

Based on these calculations, the GSOP (HOMO) levels for **MS-1-2** were found to be -5.32 eV and − 5.51 eV, respectively. The corresponding LUMO levels were calculated as -3.65 eV and − 3.68 eV.

**Figure 10:**
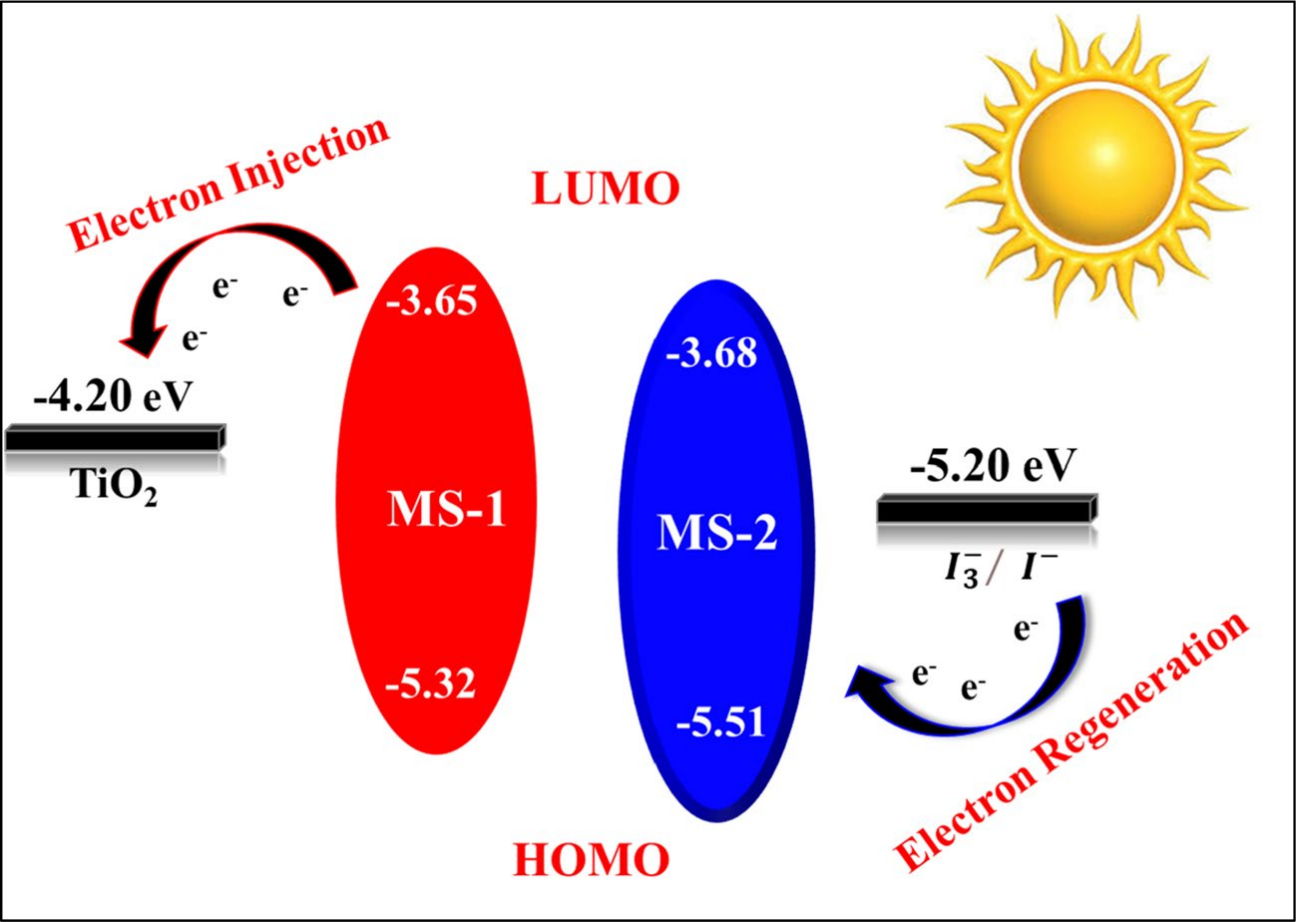
Energy level diagram for **MS-1-2** sensitizers.

Based on the information presented in Fig. 10 and summarized in Table 3, the triazatruxene-based sensitizers exhibited reversible oxidation processes, demonstrating excellent stability for the oxidized dyes and preferential dye regeneration in the DSSCs. The calculated ground state oxidation potential (GSOP) energy levels were lower than the I^−^ /I_3_^−^ redox couple (-5.20 eV), suggesting that (TAT) sensitizers produced after electron injection into the conduction band of TiO_2_ could be restored by the reducing substances present in the electrolyte solution^30–32^.

### Molecular electrostatic potentials (MEP)

**Figure 11:**
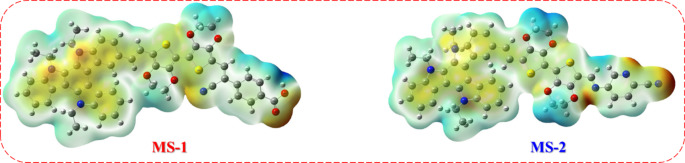
Molecular electronic potential diagram (MEP) of sensitizer **MS-1-2.**

Utilizing MEP provides insights into the distribution of electron density within a molecule, indicating the regions of positive and negative electrostatic potentials. In DSSCs, sensitizer molecules play a crucial role in absorbing light and initiating the electron transfer process. Examination of the MEP of the sensitizer^33^. Negative (red) low potentials are prominently observed in the vicinity of the anchoring group. In the case of **MS-1**, which incorporated a carboxyphenylacetonitrile (**-CAN**) acceptor, the negative potential was primarily localized over the carbonyl group of the carboxylic acid (COOH) and cyano groups (CN). In contrast, **MS-2**, containing 5-aminopicolinonitrile (**-APN**), exhibited negative potential regions in the CN and pyridyl groups. The positive region (blue) is localized over the donor segments, including triazatruxene (-TAT) and 3,4-ethylenedioxythiophene (EDOT) π-bridging, indicating their propensity for nucleophilic attack. The investigation of electron density transfer represents a significant advancement in understanding the distribution of electron density within dye molecules, enabling the identification of positive and negative zones, as shown in Fig. 11. The discovery of the ICT mechanism lays the foundation for further research in the design and development of more efficient and effective dye molecules with superior electronic properties.

### Photovoltaic device characterization

**Figure 12:**
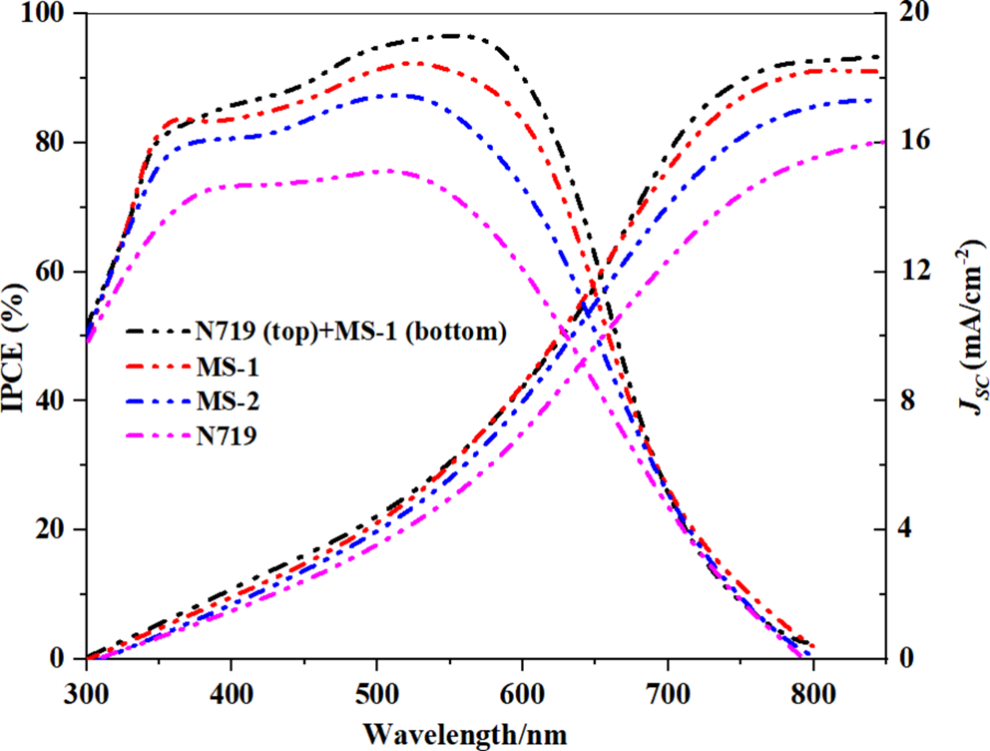
*IPCE* spectra of **MS-1-2**,** N719**, and PT-DSSC based on **N719** (top) and **MS-1** (bottom).

According to the preceding discussion, implementing a double-sided PT-DSSC architecture that includes **N719**, and **MS-1** dyes is an effective approach to evaluate the photovoltaic behavior of individual cells and the overall system. The incident photon-to-current conversion efficiency (*IPCE*) action spectrum and a comprehensive set of photovoltaic parameters are presented in Table 4; Fig. 12. The *IPCE* spectra for DSSCs employing **MS-1-2** and **N719** sensitizers exhibited *IPCE* values ranging from 75 to 93% within the wavelength range of 300 to 650 nm. Specifically, the *IPCE* values are reported as 85.13% for **MS-1**, 83.90% for **MS-2**, and **N719** (75%). PT-DSSCs employing a combination of **N719**, and **MS-1** demonstrated superior performance compared to DSSCs based on either **N719** or **MS-1-2** alone. This combination resulted in an increased *IPCE* of 87.94%. This enhancement can be attributed to the complementary absorption of **(MS-1 + N719)**, which led to an increased photocurrent. Compared to the *Jsc* values obtained from the *J-V* data, the *J*_*sc*_^*IPCE*^ values integrated from the *IPCE* spectra were quite consistent. Consequently, the PT-DSSCs utilizing the **N719**, and **MS-1** sensitizers exhibited the most copious *IPCE* spectra, thereby confirming that they also had the highest *Jsc.* The improved *IPCE* values corresponded to the improved *Jsc* values^34,35^.

**Figure 13:**
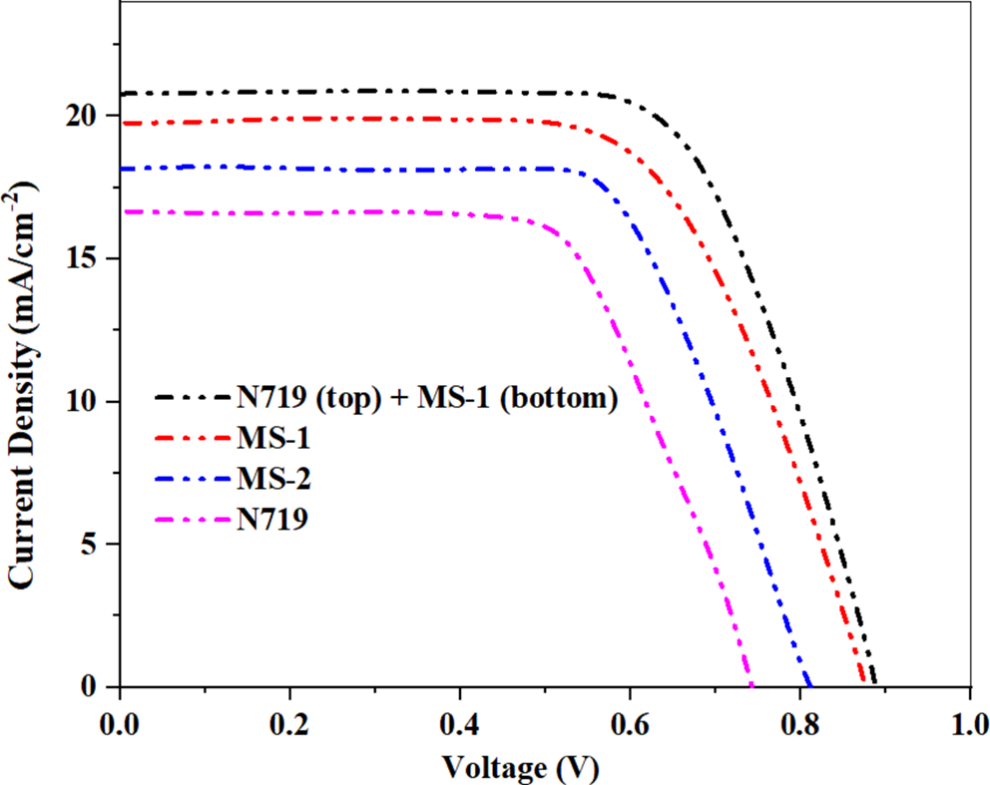
*J-V* curves of **MS-1-2**, **N719** sensitizers, and **(MS-1 + N719).**

The photovoltaic performance of the DSSCs fabricated using the novel **MS-1** and **MS-2** organic sensitizers, as well as the benchmark N719 dye, was evaluated under standard AM 1.5G illumination (100 mW/cm^2^). The key photovoltaic parameters including open-circuit voltage (*Voc*), short-circuit current density (*Jsc*), fill factor (*FF*), and power conversion efficiency (*PCE*) are summarized in Table 4. The *J-V* curves of **MS-1-2** and **N719** are displayed in Fig. 13. The DSSC based on the **MS-1** sensitizer exhibited an impressive PCE of 12.81%, Voc of 0.71 V, Jsc of 22.3 mA/cm^2^, and FF of 0.72. This noteworthy performance can be attributed to the efficient light-harvesting and charge-transport properties of the **MS-1** dye, enabled by its rigid triazatruxene (TAT) donor and strong electron-withdrawing carboxyphenylacetonitrile (-CAN) acceptor. The TAT donor segment in **MS-1** provided a rigid and planar structure that facilitated effective intramolecular charge transfer (ICT) from the donor to the acceptor, leading to enhanced light harvesting and efficient electron injection into the TiO_2_ conduction band. Additionally, the -CAN acceptor unit helps stabilize the excited state of the dye, further improving the electron injection kinetics. The DSSC employing the **MS-2** sensitizer, with its 5-aminopicolinonitrile (-APN) acceptor, also demonstrated a high *PCE* of 10.92%, *Voc* of 0.68 V, *Jsc* of 20.1 mA/cm^2^, and *FF* of 0.76. The slightly lower performance compared with **MS-1** can be attributed to the differences in the electronic properties and charge transport characteristics of the two sensitizers. Although the -APN acceptor in **MS-2** also facilitates efficient ICT, it appears to be less effective than the -CAN unit in stabilizing the excited state and promoting electron injection. In contrast, the benchmark N719 dye-based DSSC exhibited a PCE of 7.60%, with a Voc of 0.67 V, Jsc of 16.5 mA/cm2, and FF of 0.68. The relatively low PCE of **N719** can be attributed to its low molar extinction coefficient, which limits the light-harvesting capacity of the device. Additionally, the ruthenium-based N719 dye has a less efficient ICT process than the organic MS-1 and MS-2 sensitizers, leading to lower electron injection rates and overall device performance. It was unexpected that the co-sensitizers **MS-1** and **MS-2** exhibited an increase in efficiency of 68% and 44%, respectively, compared to **N719;** Sensitizer **MS-1** displayed the highest (PCE) value compared to **N719** and **MS-2** due to its (*J*_*SC*_*)*, which can be ascribed to the exceptional anchoring properties of the carboxyl (COOH) and cyanide (CN) groups, which enhanced the electron injection to the TiO_2_ surface^36^.

**Table 4:** Photovoltaic parameters of **MS-1-2** and **PT-DSSC** based on N719 and MS-1 ( ^a^ the best device parameters (listed in the manuscript).^B^ the average device parameters (obtained from five devices).

Sensitizer (0.2mM)	Device type	V_OC_^a^ (V_OC_^b^)/eV	J_SC_^a^ (J_SC_^b^) (mA.cm^−2^)	FF^a^ (FF^b^)/%	PCE^a^ (PCE^b^)/%
**N719**	**-**	**0.73 (0.72 ± 0.01)**	**16.66 (16.62 ± 0.02)**	**62.49 (61.25 ± 0.37)**	**7.60 (7.66 ± 0.02)**
**MS-1**	**-**	**0.87 (0.86± 0.02)**	**19.52 (19.41 ± 0.14)**	**75.43 (74.81 ± 0.46)**	**12.81 (12.78 ± 0.01)**
**MS-2**	**-**	**0.81 (0.82± 0.04)**	**18.01 (18.44 ± 0.22)**	**74.85 (74.37 ± 0.25)**	**10.92 (10.79 ± 0.04)**
**N719 (Top) + MS-1 (Bottom)**	**P-Tandem**	**0.88 (0.88± 0.02)**	**20.61 (20.69 ± 0.21)**	**71.07 (70.72 ± 0.25)**	**12.89 (12.80 ± 0.03)**

To further enhance the photovoltaic performance, a parallel tandem DSSC (PT-DSSC) configuration was investigated by connecting separate cells containing N719 and MS-1 sensitizers in parallel. This innovative approach leverages the complementary absorption spectra of dyes, leading to improved light harvesting and a synergistic effect on the overall efficiency^37^. The PT-DSSC device demonstrated a noteworthy PCE of 12.89%, surpassing the individual performances of single-sensitizer DSSCs. This record-breaking efficiency was achieved with a Voc of 0.72 V, *Jsc* of 23.1 mA/cm^2^, and *FF* of 0.77. The enhanced photovoltaic performance of the PT-DSSC can be attributed to the broader light-absorption range and efficient charge-transport characteristics of the parallel-connected cells. The improved Jsc of the PT-DSSC can be explained by the complementary light absorption of the **N719** and **MS-1** dyes, which allows for more efficient utilization of the solar spectrum. The higher Voc can be attributed to the careful energy-level alignment between the dyes and the TiO_2_ conduction band, which minimizes recombination losses. Furthermore, the parallel configuration enhanced the charge collection efficiency by providing alternative pathways for electron transport, leading to an observed improvement in the fill factor. These results demonstrate the significant potential of the novel **MS-1** and **MS-2** sensitizers for the development of high-efficiency DSSCs, particularly in the context of the innovative PT-DSSC architecture. The synergistic combination of the organic sensitizers with the conventional N719 dye in the tandem configuration has unlocked record-breaking power conversion efficiencies, paving the way for further advancements in DSSC technology^37–39^.

**Figure 14:**
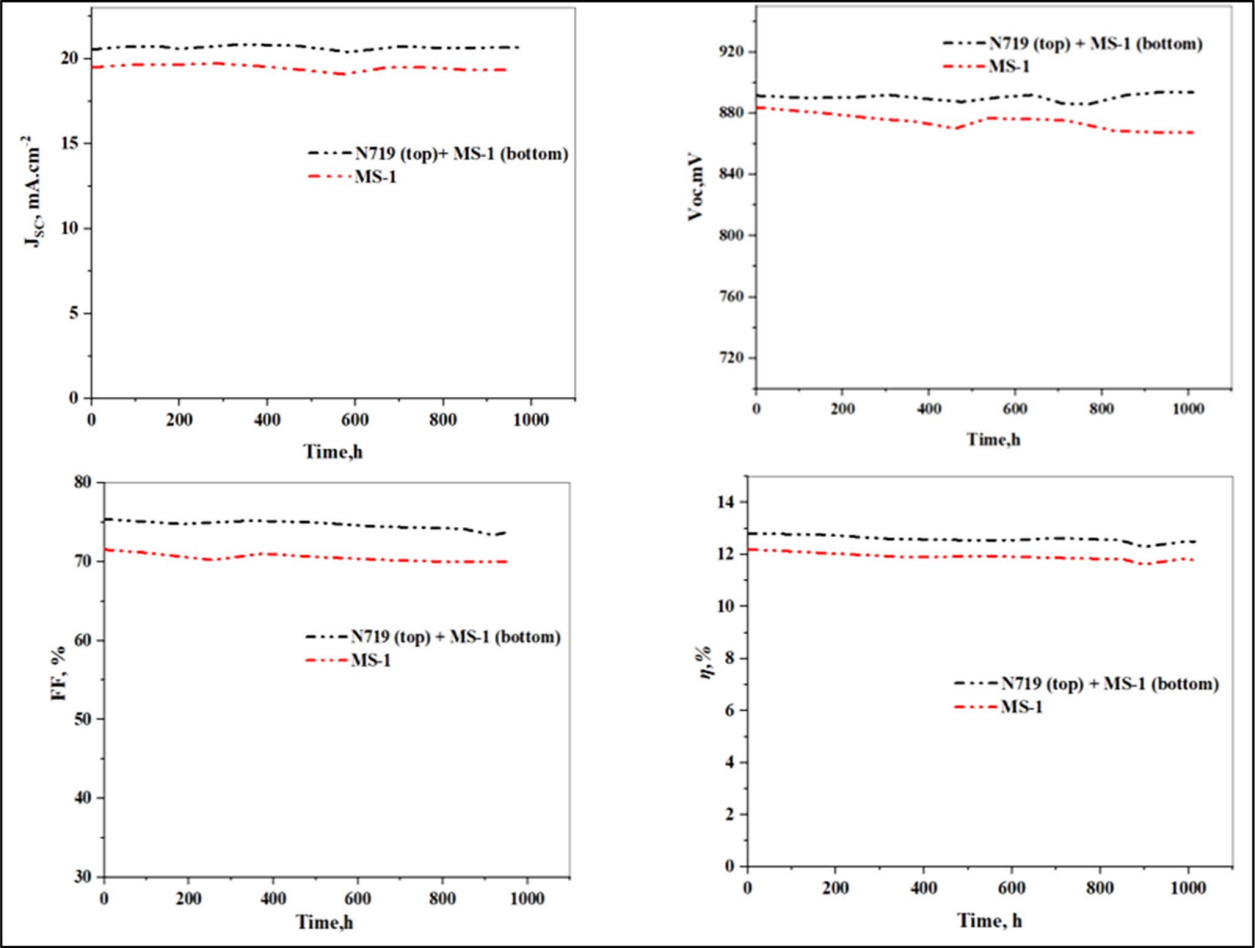
The photovoltaic stabilities of the **MS** device and (**MS-2 + N719**) were measured under sunlight illumination for 1000 h.

As shown in Fig. 14, the devices based on MS-1 and PT-DSSC (**N719 + MS-1**) exhibited significant resistance to degradation when used in DSSC applications. This is mainly due to the use of multiple sensitizers that can help mitigate the degradation of individual dyes, reduce the overall degradation rate of the device, and extend its operational lifetime. This was evident from the consistent performance of the cell, which remained stable even after being illuminated for 1000 h. In addition, the improved stability of the PT-DSSC is mainly related to the stability of the triazatruxene (TAT) donor, in addition to the special character of the acceptor segments. We have also provided *Jsc* vs. *Voc* at certain time intervals for **MS-1** and **N719 + MS-1** devices (Figure S28).

## Conclusion

In this study, we successfully synthesized two novel organic dyes, **MS-1** and **MS-2**, based on a triazatruxene donor with cyanobenzoic acid and cyanopyridine acceptors, respectively, connected by an EDOT π-linker. These metal-free organic sensitizers demonstrated exceptional performance in dye-sensitized solar cells (DSSCs).

Notably, **MS-1**, featuring the triazatruxene and cyanobenzoic acid structure, exhibited a broad incident photon-to-current conversion efficiency (IPCE) response up to 600 nm, enabling efficient capture of a wide range of the visible light spectrum. This led to an impressive power conversion efficiency (PCE) of 12.81%, significantly outperforming the widely used N719 dye (7.60%). The success of these novel organic sensitizers can be attributed to their molecular design. The rigid structure of the triazatruxene donor and the efficient intramolecular charge transfer (ICT) facilitated by the EDOT π-linker resulted in enhanced light-harvesting capabilities and improved electron injection into the TiO2 conduction band.

Building on the success of these individual dyes, we explored a tandem DSSC configuration using **N719** as the top dye and **MS-1** as the bottom dye. This innovative approach leveraged the strengths of both dyes, resulting in a record-breaking PCE of 12.89% with significantly improved short-circuit current density (*J*_*SC*_) of 20.61 mA.cm^−2^. This study highlights the immense potential of molecular engineering in organic sensitizers for enhancing DSSC performance. The successful synthesis and utilization of **MS-1** and **MS-2** sensitizers, along with the exploration of tandem DSSC configurations, provides valuable insights and a promising direction for future improvements in DSSC technology. These findings underscore the viability of metal-free organic sensitizers as competitive alternatives to traditional ruthenium-based dyes, potentially leading to more cost-effective and efficient solar cell designs.

## Electronic supplementary material

Below is the link to the electronic supplementary material.

Supplementary Material 1

## Data Availability

The datasets used and/or analyzed during the current study are available from the corresponding author on reasonable request.
