# Supplementary material for: Tandem dye-sensitized solar cells achieve 12.89% efficiency using novel organic sensitizers
Source: Sci Rep. 2024 Oct 30;14:26072. doi: 10.1038/s41598-024-75959-0 (PMC11525810; doi:10.1038/s41598-024-75959-0)
Supplement: Supplementary file 1 — Supplementary Material 1 [file 41598_2024_75959_MOESM1_ESM.docx]

**Supplementary file**

**Tandem Dye-Sensitized Solar Cells Achieve 12.89% Efficiency Using Novel Organic Sensitizers**

Safa A. Badawy*^🖂^*, Ehab Abdel-Latif, Mohamed R. Elmorsy *^🖂^*

*Department of Chemistry, Faculty of Science, Mansoura University, 35516 Mansoura, Egypt.*

*^🖂^Corresponding author:* [*m.r.elmorsy@mans.edu.eg*](mailto:m.r.elmorsy@mans.edu.eg)***,*** [*safabadawy140@gmail.com*](mailto:safabadawy140@gmail.com)

***1. Materials and methods***

The necessary chemicals and solvents for the synthetic procedures were procured from reputable sources such as Sigma-Aldrich, and Alfa Aesar, and used without modification. The measured melting points, reported in degrees Celsius, were obtained using a Gallenkamp electric melting point instrument and are uncorrected. IR spectra (KBr) were obtained using a Thermo Scientific Nicolet iS10 FTIR spectrometer, while NMR spectra were acquired in DMSO-d6 using a Burker NMR spectrometer operating at frequencies of 400 MHz (^1^H NMR) and 100 MHz (^13^C NMR), respectively. UV-Visible spectra were collected utilizing a high-performance double beam spectrophotometer (T80 series). Mass analyses were recorded using a Thermo DSQ II spectrometer, while the elemental analysis data were obtained using a Perkin Elmer 2400 analyzer. Detailed information regarding the instruments and DSSC fabrications utilized can be found in the accompanying information file.

**2. Synthetic details and characteristic results for all the compounds**

***2.1. 5,6,11-Triethyl-6,11-dihydro-5H-diindolo[2,3-a:2',3'-c]carbazole (1):***

Phosphorous (V) oxychloride (POCl_3_) (10 mL) and 2-oxoindole (2 g, 0.015 mol) were added into a 250 mL round-bottomed flask and the reaction mixture was stirred at 150 °C for 16 h. The solution was cooled to room temperature and transferred into a 500 mL beaker containing crushed ice (250 mL) and neutralized by the addition of adequate amount of saturated NaOH solution. The resulting brown colored precipitate was collected by filtration and the crude product was purified by recrystallization by mixture of ethanol/acetone solvent. Synthesis and Characterization of 5,6,11-Triethyl-6,11-dihydro-5H-diindolo[2,3-a:2',3'-c]carbazole (TAT-ALKYL) TAT (0.5 g, 1.45 mmol) and NaH (0.81 g, 14.50 mmol) was dissolved in 100 mL of anhydrous DMF heated at 70 °C for 3.5 h. After the mixture was cooled to room temperature, ethyl iodide (0.65 g, 4.78 mmol) was added to mixture. The mixture was stirred magnetically at room temperature for 24 hr. After the reaction was completed, the reaction poured into ice crushed water. The crude product was purified by silica gel column chromatography by using 1:4 CH_2_Cl_2_/hexane. After crystallization over 1:1 CH_2_Cl_2_/acetone. ^1^H NMR (DMSO*-d_6_*): *δ* 1.35-1.41 (m, 9H, -CH_2_), 4.06 (q, *J* = 8.00 Hz, 2H, -CH_2_), 4.53 (q, *J* = 8.00 Hz, 2H, -CH_2_), 5.17 (q, *J* = 8.00 Hz, 2H, -CH_2_), 7.22-7.25 (m, 6H, Ar-H), 7.34-7.38 (m, 3H, Ar-H), 7.58 (d, *J* = 8.00 Hz, 1H, Ar-H), 7.67 (d, *J* = 8.00 Hz, 1H, Ar-H), 7.72 (d, *J* = 8.00 Hz, 1H, Ar-H). ^13^C NMR (DMSO-*d*_6_): *δ* 13.33 (3C), 43.92 (3C), 111.62 (2C), 112.33, 115.53, 116.72, 122.14 (3C), 123.65, 123.90 (3C), 124.88, 125.69 (2C), 126.69 (3C), 128.48, 129.41, 140.31, 141.12, 145.64 (2C) ppm. Analysis calcd. for C_30_H_27_N_3_(429.57): C, 83.88; H, 6.34; N, 9.78%. Found: C,83.76; H, 6.44; N, 9.89%.

***2.2. 5,6,11-Triethyl-6,11-dihydro-5H-diindolo[2,3-a:2',3'-c]carbazole-2-carbaldehyde (2).***

In a 100 mL three-neck RB flask, 2.89 mL of dry DMF (37.35 mmol) was added to 3.49 mL of freshly distilled POCl_3_ (23.40 mmol) in an atmosphere of N_2_ nitrogen at 0°C. The resulting solution was stirred until it was completely converted to a Vilsmeier glassy solid salt. Subsequently, to 30 ml dry DMF, (4.29 g, 10 mmol) of triazatruxene (**1**) was added dropwise to 30 mL of dry DMF, and the reaction mixture was stirred for 48 h at 50 ^0^C and poured into 250 mL of ice-cold crushed water. The crude product was recrystallized from a mixture of ethanol and petroleum ether, yielding a yellowish product in 78 % yield. IR (KBr): *ν_max_* 2914, 2789 (C-H), 1648 (C=O) and (C=C) 1600 cm^-1^. ^1^H NMR (DMSO*-d_6_*): *δ* 1.34-1.41 (m, 9H, 3CH_3_), 4.23 (q, *J* = 6.50 Hz, 2H, CH_2_), 4.54 (q, *J* = 6.50 Hz, 2H, CH_2_), 4.77 (q, *J* = 6.50 Hz, 2H, CH_2_), 7.23-7.26 (m, 5H, Ar-H), 7.35-7.39 (m, 2H, Ar-H), 7.53 (d, *J*=8.00 Hz, 1H, Ar-H), 7.57-7.59 (m, 1H, Ar-H), 7.63-7.66 (m, 1H, Ar-H), 7.78 (s, 1H, *J*=8.00 Hz, Ar-H), 8.63 (s, 1H, CHO). ^13^C NMR (DMSO-*d*_6_): *δ* 15.75 (3C), 46.35 (3C), 111.82, 114.05, 114.76, 117.99, 118.99, 124.45, 124.51, 124.57, 126.33, 127.05, 128.12, 128.32, 128.79 (3C), 129.25, 130.91, 132.59, 134.53, 136.92, 143.34, 143.55, 148.07, 150.67, 193.10 ppm. Elemental Analysis calculated. for C_31_H_27_N_3_O (457.22): C, 81.37; H, 5.95; N, 9.18 %. Found: C, 81.62; H, 5.83; N, 8.98 %.

***2.3.(5,6,11-Triethyl-6,11-dihydro-5H-diindolo[2,3-a:2',3'-c]carbazol-2-yl)methanol (3):***

In a 100 mL two-neck flask, compound 2 (0.45 g, 0.10 mmol) was dissolved in methanol (80 mL)/ dichloromethane (CH_2_Cl_2_) (20 mL). Under argon protection, add NaBH_4_ (0.3 g) to the two-necked flask. The reaction was stirring for 12 hours. After stopping the reaction, pour on into ice crushed ice and extract with ethyl acetate and collect the organic phase. Dry the organic phase with Na_2_SO_4_. The compound **4** was collected to obtain a white solid crystal, with melting point 70 °C. ^1^H NMR (DMSO*-d_6_*): *δ* 1.33-1.37 (m, 9H, 3CH_3_), 4.53 (q, *J* = 6.50 Hz, 2H, CH_2_), 4.76 (q, *J* = 8.00 Hz, 2H, CH_2_), 4.96 (q, *J* = 8.00 Hz, 2H, CH_2_), 5.17 (s, 2H, CH_2_), 5.73 (s, 1H, -OH), 7.18 (d, 1H, *J* =8.00 Hz, Ar-H), 7.23-7.26 (m, 4H, Ar-H), 7.34-7.38 (m, 3H, Ar-H), 7.57 (d, 1H, *J*=8.00 Hz, Ar-H), 7.68 (d, 1H, *J*=8.00 Hz, Ar-H), 7.73 (s, 1H, Ar-H). ^13^C NMR (DMSO-*d*_6_): *δ* 14.44 (3C), 45.03 (3C), 64.64, 112.74, 112.91, 113.44, 116.67, 117.67, 123.13, 123.19, 123.25, 125.01, 125.60, 125.73, 126.80, 127.31, 127.48 (3C), 128.77, 129.59, 131.27, 137.62, 142.02, 142.23, 145.40, 146.75 ppm. Elemental Analysis calculated. For C_31_H_29_N_3_O: C, 81.02; H, 6.36; N, 9.14%. Found: C, 81.17; H, 6.29; N, 9.25 %.

***2.4. 2-((Bromotriphenyl-λ^5^-phosphaneyl)methyl)-5,6,11-triethyl-6,11-dihydro-5H-diindolo[2,3-a:2',3'-c]carbazole (4):***

In a 100 mL two-neck flask, alcohol compound 3 (0.18 g, 0.40 mmol) and triphenylphosphine hydrobromide (PPh_3_HBr) (0.38 g, 1.12 mmol) were dissolved in chloroform (CHCl_3_) (100 mL). The reaction was stirred for 2hr, then temperature was raised to 80°C and the reaction was stirred with refluxed for 30 h and followed by TLC until the reaction finished. After fishing the reaction, cool to room temperature, then pour into ice cold water. Extract with EA and collect the organic phase. Dry the organic phase with Na_2_SO_4_. After mixing the silica gel powder and drying it, the organic phase is separated and purified by silica gel column chromatography (Hexane: MeOH = 10:1, v:v). The target phosphine salt **4** was collected to obtain a white solid.

^1^H NMR (DMSO*-d_6_*): *δ* 1.35-1.42 (m, 9H, 3CH_3_), 2.51(s, 2H, CH_2_), 4.06 (q, *J* = 8.00 Hz, 2H, CH_2_), 4.55-4.60 (m, 4H, CH_2_), 7.00 (d, *J* = 8.00 Hz, 2H, Ar-H), 7.10 (t, *J* = 8.00 Hz, 2H, Ar-H), 7.18-7.25 (m, 6H, Ar-H), 7.30 (t, *J* =8.00 Hz, 2H, Ar-H), 7.42 (t, *J* = 8.00 Hz, 4H, Ar-H), 7.54-7.63 (m, 9H, Ar-H), 7.72 (s, 1H, Ar-H). ^13^C NMR (DMSO-*d*_6_): *δ* 13.33 (3C), 29.79, 43.92 (3C), 111.62, 112.33, 113.27, 115.56, 116.56, 118.96 (3C), 122.01, 122.14, 123.38, 123.90 (2 C), 124.62, 125.69, 126.37 (3C), 128.05, 128.48, 128.62, 130.15, 131.58, 132.87 (5C), 133.81(5C), 134.88 (5C), 140.91, 141.12, 144.27, 145.64 ppm.

***2.5. 2,2',3,3'-Tetrahydro-[5,5'-bithieno[3,4-b][1,4]dioxine]-7,7'-dicarbaldehyde (5):***

In a 100 mL two-neck round-bottom flask, 20 ml of dry dimethylformamide (DMF) was stirred under an argon atmosphere at 0°C. Subsequently, 15 mL of freshly (POCl_3_) was added dropwise until the colored Vilsmeier salt was completely precipitated. The resulting reaction mixture was stirred continuously for 1 h, during which time a solution of compound **(5)** (1.5 g of 2,2',3,3'-tetrahydro-5,5'-bithieno[3,4-b][1,4]dioxine dissolved in 15 mL of dichloromethane was slowly added dropwise. Following this addition, the temperature was raised to 80°C and held for 2 h before the mixture was stirred at room temperature overnight. After completion of the reaction, the mixture was immersed in ice-cold water. The pH was increased to an alkaline level by adding a saturated sodium acetate solution, which resulted in the synthesis of a yellow powdered compound the . ^1^H NMR (DMSO*-d_6_*): *δ* 4.36 (br, s, 4H, -CH_2_), 4.93 (brs, 4H, -CH_2_), 9.64 (s, 2H, CHO) ppm. ^13^C NMR (DMSO-*d*_6_): *δ* 66.72 (4C), 111.63 (2C), 122.34 (2C), 135.90 (2C), 155.19 (2C), 187.17 (2C) ppm. Analysis calcd. for C_14_H_10_O_6_S_2_ (338.35): C, 49.70; H, 2.98 %. Found: C, 49.79; H, 2.87%.

***2.6. 7'-(2-(5,6,11-Triethyl-6,11-dihydro-5H-diindolo[2,3-a:2',3'-c]carbazol-2-yl)vinyl)-2,2',3,3'-tetrahydro-[5,5'-bithieno[3,4-b][1,4]dioxine]-7-carbaldehyde (6)***

TAT-aldehyde was synthesized via two reactions. The first reaction involved the formation of a phosphonium salt of 5,6,11-triethyl-6,11-dihydro-5*H*-diindolo[2,3-a:2',3'-c]carbazole and bi-EDOT dialdehyde (**5**). The Wittig reaction was carried out under alkaline conditions (t-BuOK) and a N_2_ nitrogen atmosphere at 0^ο^C. The reaction mixture was refluxed with continuous stirring at 70°C for 30 h. Subsequently, the mixture was poured into ice-cold water, neutralized with sodium carbonate, and extracted with ethyl acetate. The organic layer was then separated and dried over anhydrous sodium sulfate (NaSO_4_). This process yielded a yellowish oily product in 70% yield.

IR (KBr): *ν_max_* 2959, 2893, 2828 (C-H), 1714 (C=O), (C=C) 1613 cm^-1^. ^1^H NMR (DMSO*-d_6_*): *δ* 1.37-1.40 (m, 8H, -CH_2_), 4.21-4.24 (m, 4H, -CH_2_), 4.30-4.36 (m, 10H, -CH_2_), 7.03 (t, *J* = 12.00 Hz, 1H, =CH-H), 7.28-7.40 (m, 8H, Ar-H), 7.54 (s, 1H, =CH), 8.03 (d, *J* = 8.00 Hz, 2H, Ar-H), 8.08 (d, *J* = 8.00 Hz, 1H, Ar-H), 8.43 (d, *J* = 8.00 Hz, 1H, Ar-H), 9.13 (s, 1H, CHO) ppm. ^13^C NMR (DMSO-*d*_6_): *δ* 17.33 (3C), 47.92 (3C), 58.53 (2C), 70.72 (2C), 112.54, 115.63 (2C), 116.33, 119.56 (2C), 120.56 (2C), 125.73, 126.01, 126.08, 126.14, 126.34, 127.27, 127.90, 128.23, 128.62, 129.68, 130.37 (4C), 132.48, 134.15, 136.24, 136.38, 136.52, 139.90, 144.91, 145.12, 147.11, 149.64, 155.02, 159.19, 191.17 ppm. Analysis calcd. for C_45_H_37_N_3_O_5_S_2_ (763.93): C, 70.75; H, 4.88; N, 5.50 %. Found: C, 70.54; H, 4.97; N, 5.38%.

***2.7. 4-(1-Cyano-2-(7'-((E)-2-(5,6,11-triethyl-6,11-dihydro-5H-diindolo[2,3-a:2',3'-c]carbazol-2-yl)vinyl)-2,2',3,3'-tetrahydro-[5,5'-bithieno[3,4-b][1,4]dioxin]-7-yl)vinyl)benzoic acid (MS-1)***

Compound **6** (7.63 g, 0.01 mol) and 4-(cyanomethyl)benzoic acid (1.61 g, 0.012 mol) were dissolved in methanol (100 mL) under a nitrogen atmosphere. Piperidine (0.2 mL) was added as the catalyst, and the reaction mixture was refluxed at 100°C for 12 h. After cooling to room temperature, the solid precipitate was filtered, washed with ethanol, dried, and recrystallized from a 10:2 ethanol/acetic acid mixture to obtain a purple solid with a melting point of 280-282°C. IR (KBr): *ν_max_* 2995, 2920 (C-H), 2207 (C≡N), 1687 (C=O), 1619 cm^-1^ (C=C). ^1^H NMR (DMSO*-d_6_*): δ 1.36-1.39 (m, 9H, CH_2_), 4.21- 4.24 (m, 8H, CH_2_), 4.30-4.36 (m, 6H, -CH_2_), 7.03 (d, *J* = 12.00 Hz, 2H, Ar-H), 7.28-7.34 (m, 2H, Ar-H), 7.37-7.40 (m, 4H, Ar-H), 7.54 (s, 1H, =CH), 7.62 (d, *J* = 8.00 Hz, 2H, Ar-H), 7.68 (d, *J* = 8.00 Hz, 2H, Ar-H), 8.03 (d, *J* = 8.00 Hz, 2H, Ar-H), 8.08 (d, *J* = 8.00 Hz, 1H, Ar-H), 8.23 (d, *J* = 8.00 Hz, 1H, Ar-H), 8.43 (d, *J* = 8.00 Hz, 1H, Ar-H) ppm. ^13^C NMR (DMSO-*d*_6_): *δ* 13.33 (3C), 43.92 (2C), 57.01 (2C), 66.72 (2C), 107.86, 108.57, 111.63 (5C), 112.33, 115.56 (2C), 116.56, 118.16, 121.73, 122.01, 122.08, 122.14, 123.27, 123.90, 124.23, 124.62, 125.12 (2C), 125.69, 126.37 (2C), 128.14, 128.48, 128.75 (2C), 130.15 (2C), 132.24, 132.38, 132.52, 133.69, 135.90 (2C), 140.91, 141.12, 143.11, 145.64, 149.65, 151.02, 168.95 ppm. Analysis calcd. for C_54_H_42_N_4_O_6_S_2_ (907.07): C, 71.50; H, 4.67; N, 6.18 %. Found: C, 71.78; H, 4.82; N, 6.06 %.

***2.8. 5-(((E)-(7'-((E)-2-(5,6,11-Triethyl-6,11-dihydro-5H-diindolo[2,3-a:2',3'-c]carbazol-2-yl)vinyl)-2,2',3,3'-tetrahydro-[5,5'-bithieno[3,4-b][1,4]dioxin]-7-yl)methylene)amino)picolinonitrile (MS-2)***

In 100 ml of ethanol solution, TAT-aldehyde **(6)** (0.763 g, 1 mmol) and 5-aminopicolinonitrile (1.66 g, 14 mmol) in was stirred and refluxed for 28 hours under nitrogen atmosphere in the presence of a basic catalytic of (DBU) 1,8-diazabicyclo[5.4.0]undec-7-ene. The resulting sensitizer was precipitated by cooling it to room temperature. It was then filtered, washed thoroughly with ethanol, and isolated to obtain **MS-2** sensitizer.

m.p. = 224-226°C. IR (KBr): *ν_max_* 2924, 2841 (-CH), 2211 (C≡N), 1620 cm^-1^ (C=C).

^1^H NMR (DMSO*-d_6_*): *δ* 1.36-1.39 (m, 9H, CH_2_), 4.21-4.24 (m, 8H, CH_2_), 4.30-4.36 (m, 6H, -CH_2_), 7.05 (d, *J* = 12.00 Hz, 1H, Ar-H), 7.28-7.31 (m, 2H, Ar-H), 7.54 (s, 1H, =CH), 7.37-7.40 (m, 4H, Ar-H), 7.68-7.71 (m, 1H, Ar-H), 7.81 (d, *J* = 8.00 Hz, 2H, Ar-H), 8.08 (d, *J* = 8.00 Hz, 2H, Ar-H), 8.23-8.25 (m, 1H, Ar-H), 8.45 (d, *J* = 4.00 Hz, 1H, Py-H), 8.66 (s, 1H, =CH), 8.74 (d, *J* = 4.00 Hz, 1H, Py-H) ppm. ^13^C NMR (DMSO-*d*_6_): *δ* 14.34 (3C), 44.94 (3C), 54.72 (2C), 67.73 (2C), 109.56, 112.65, 113.34, 116.58, 117.58, 117.70, 121.13, 122.74, 123.03, 123.09, 123.15, 124.28, 124.91, 124.97 (2C), 125.25, 125.63, 126.70, 127.39 (3C), 128.03 (2C), 129.50, 131.07(2C), 131.17, 133.26, 133.39, 133.54, 136.91, 141.92, 142.14, 143.82 (2C), 144.13, 146.65, 149.65, 149.68, 150.10, 152.03 ppm. Analysis calcd. for C_51_H_40_N_6_O_4_S_2_ (865.04): C, 70.81; H, 4.66; N, 9.72 %. Found: C, 71.02; H, 4.78; N, 9.84%.

**3. List of Figures:**


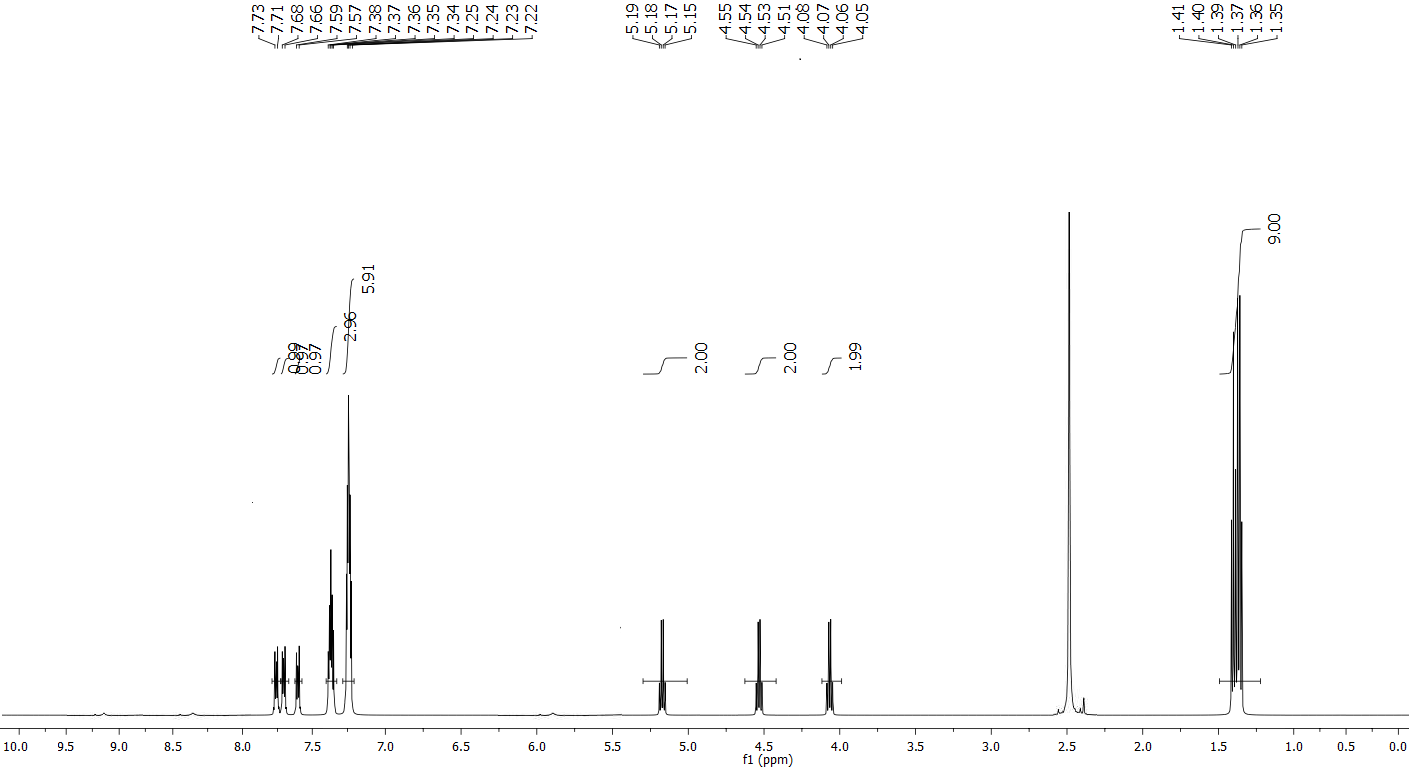


**Figure (S1): ^1^H NMR spectrum of compound** 1.


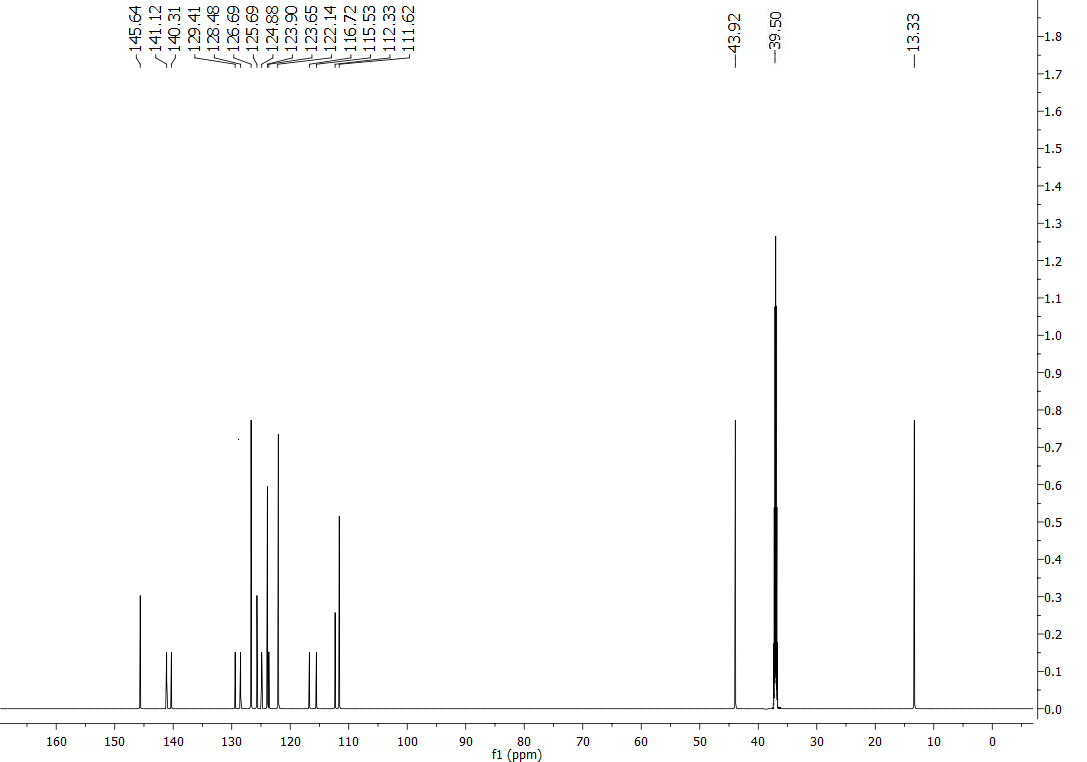


**Figure (S2): ^13^CNMR spectrum of compound** 1.


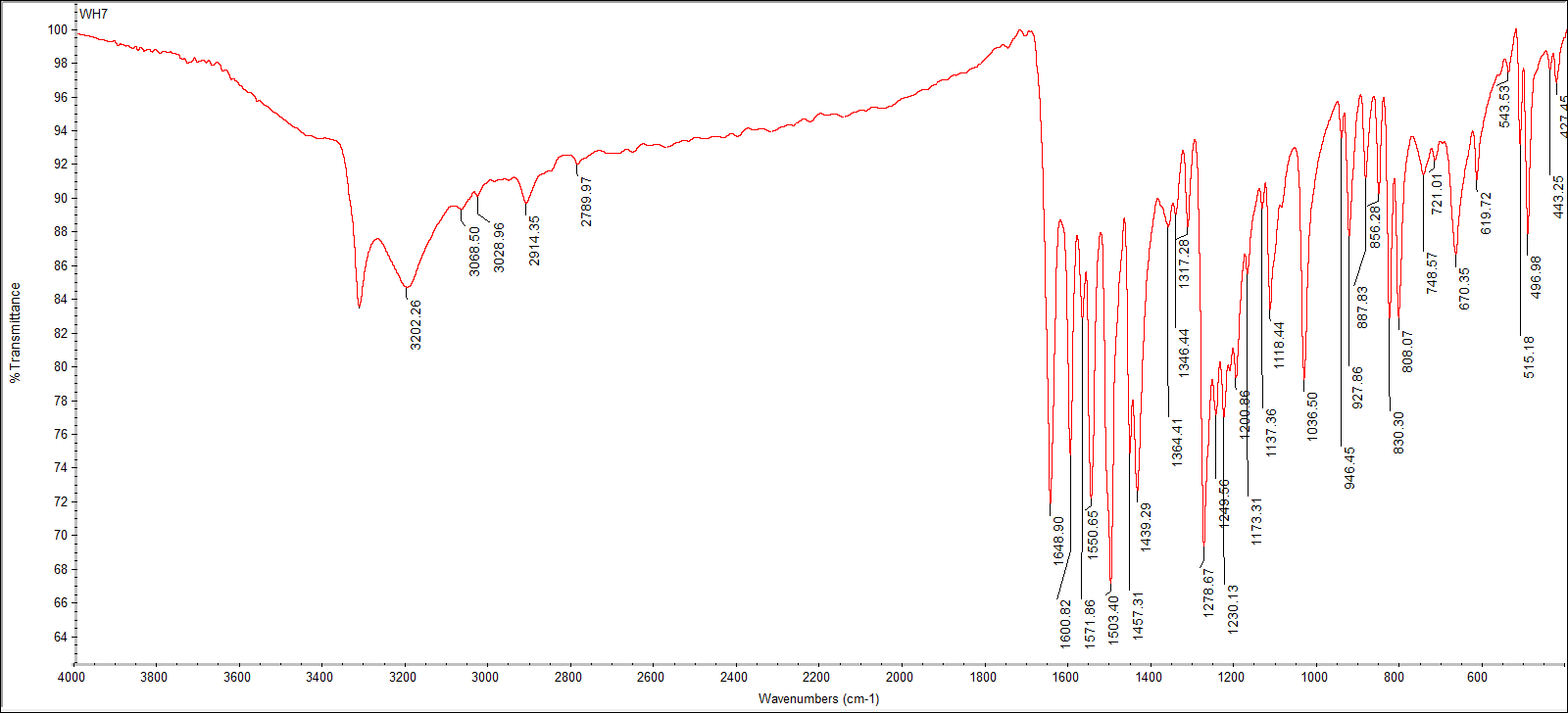

**Figure (S3): IR spectrum of compound 2.**


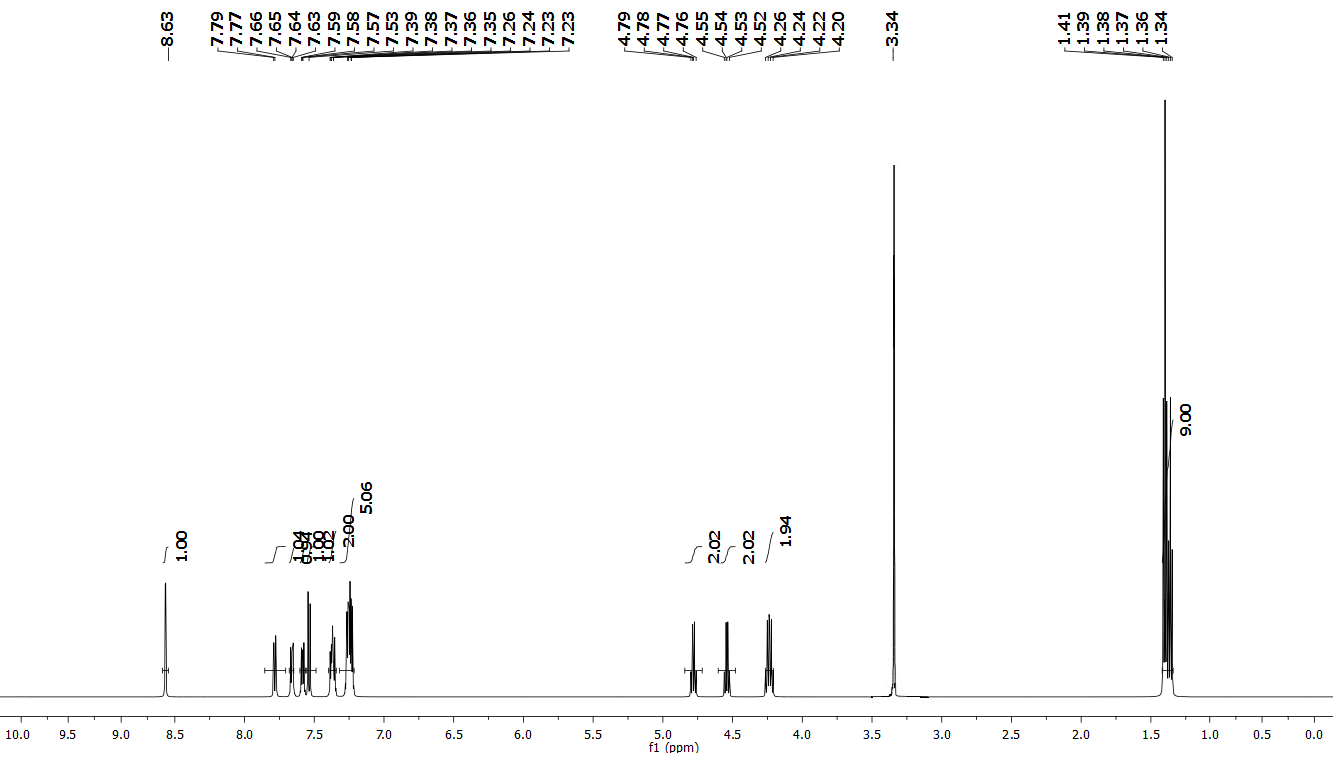

**Figure (S4): ^1^H NMR spectrum of compound 2.**


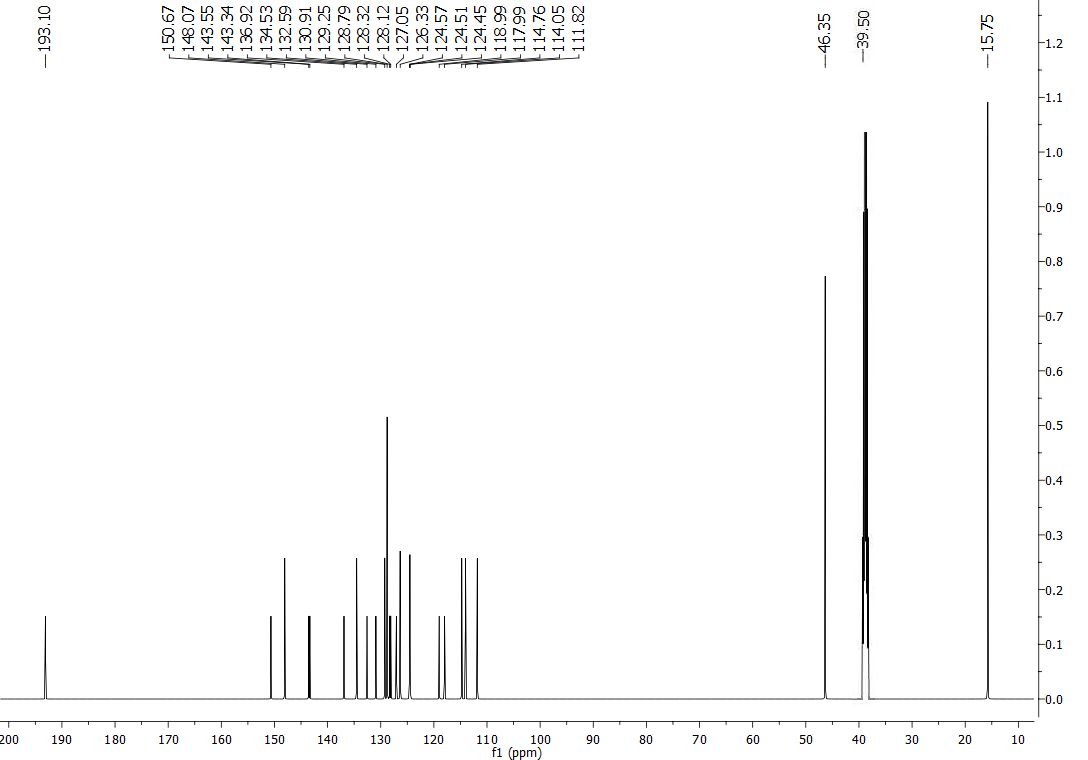

**Figure (S5): C^13^ NMR spectrum of compound 2.**


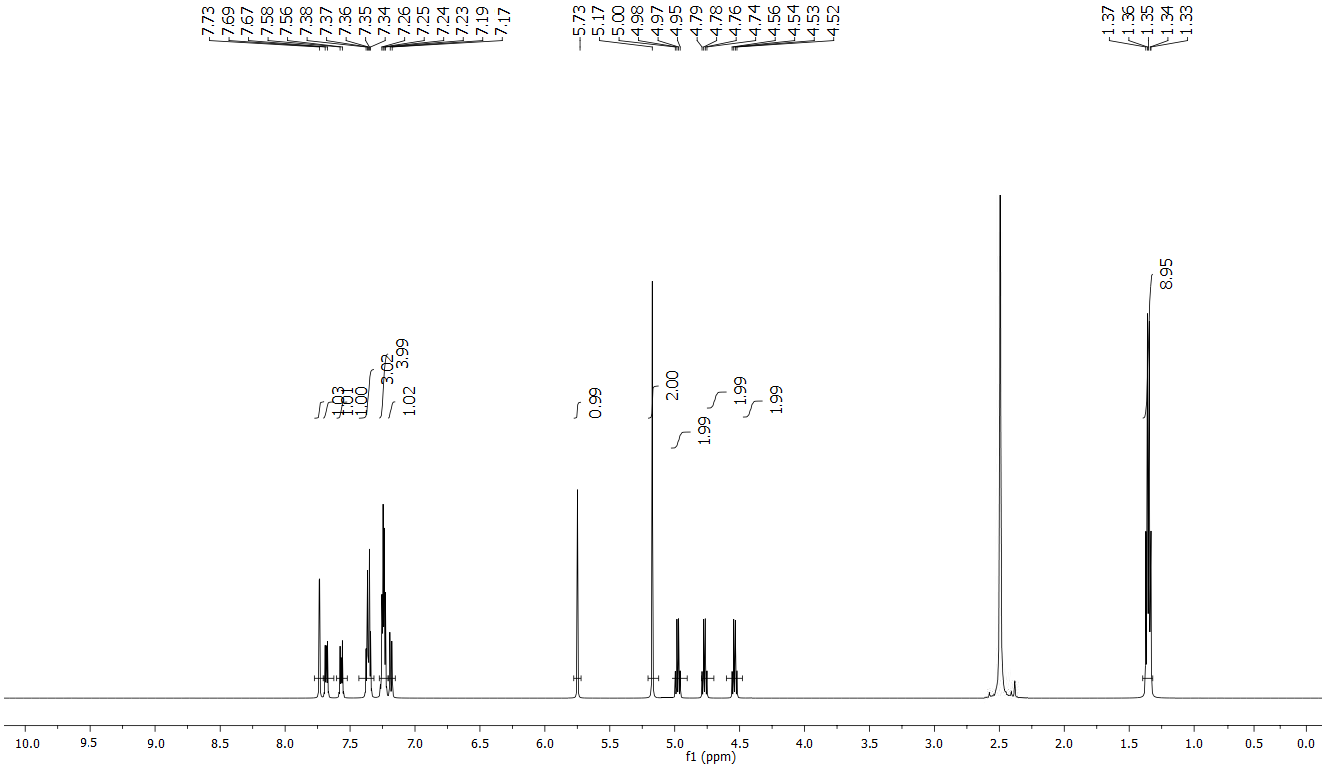


**Figure (S6): ^1^H NMR spectrum of compound 3.**


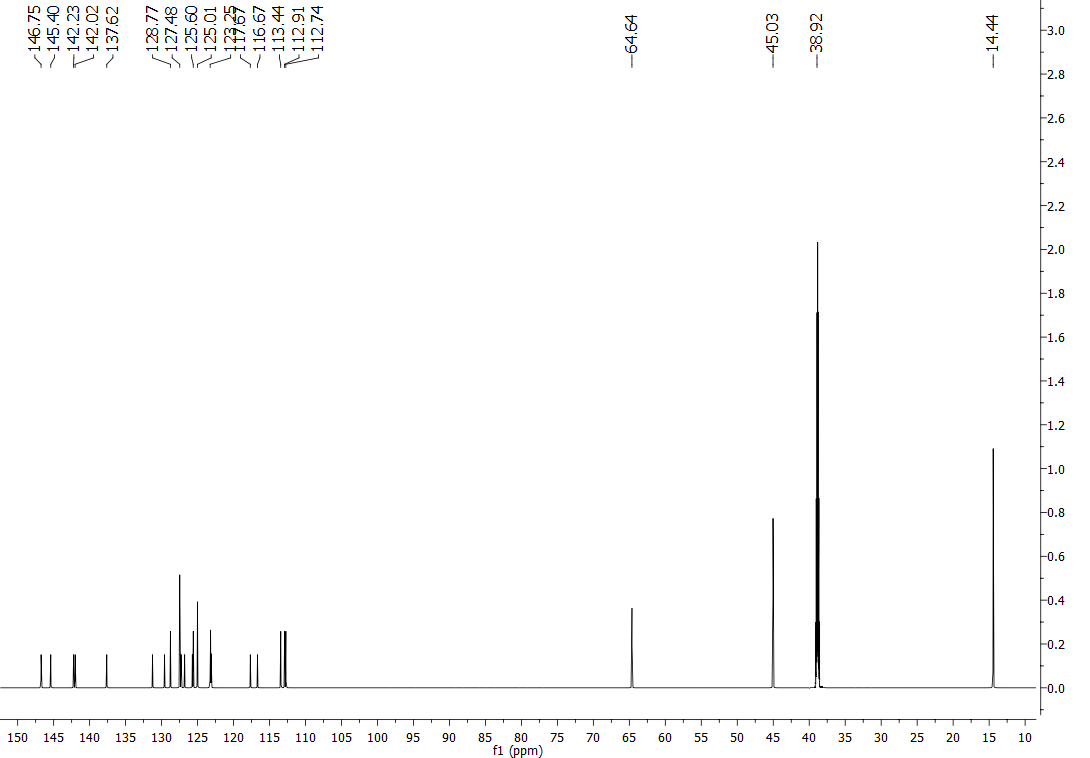


**Figure (S7): C^13^ NMR spectrum of compound 3.**


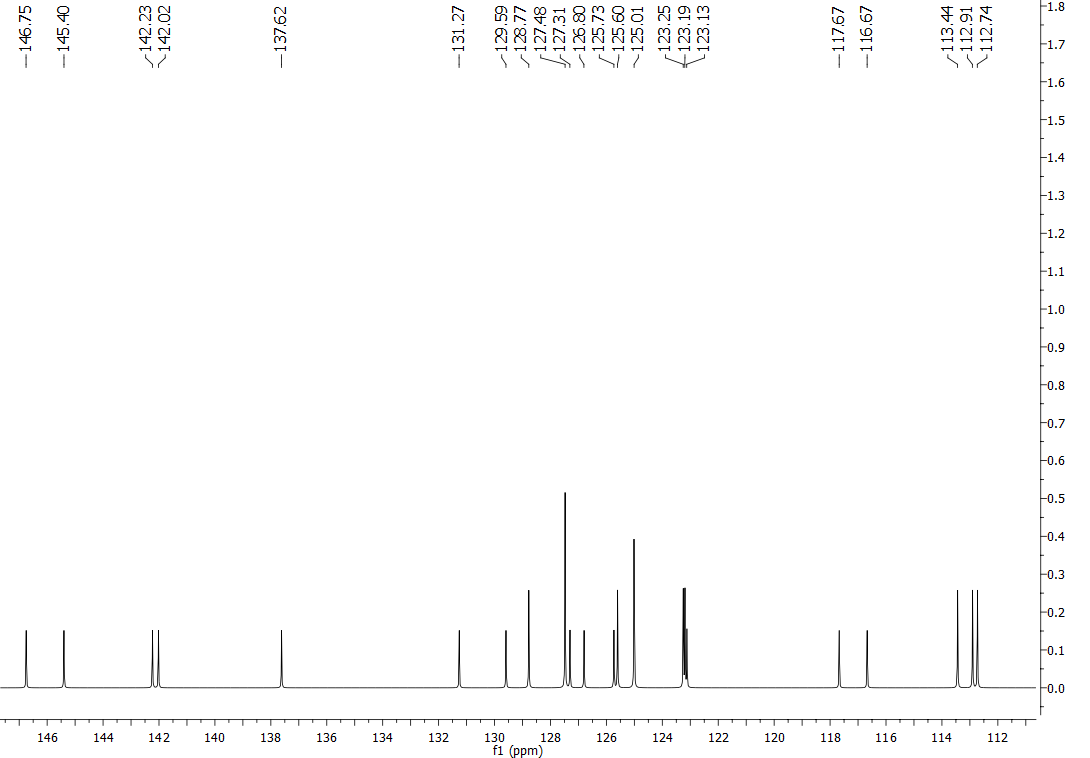


**Figure (S8): Zoom C^13^ NMR spectrum of compound 3.**


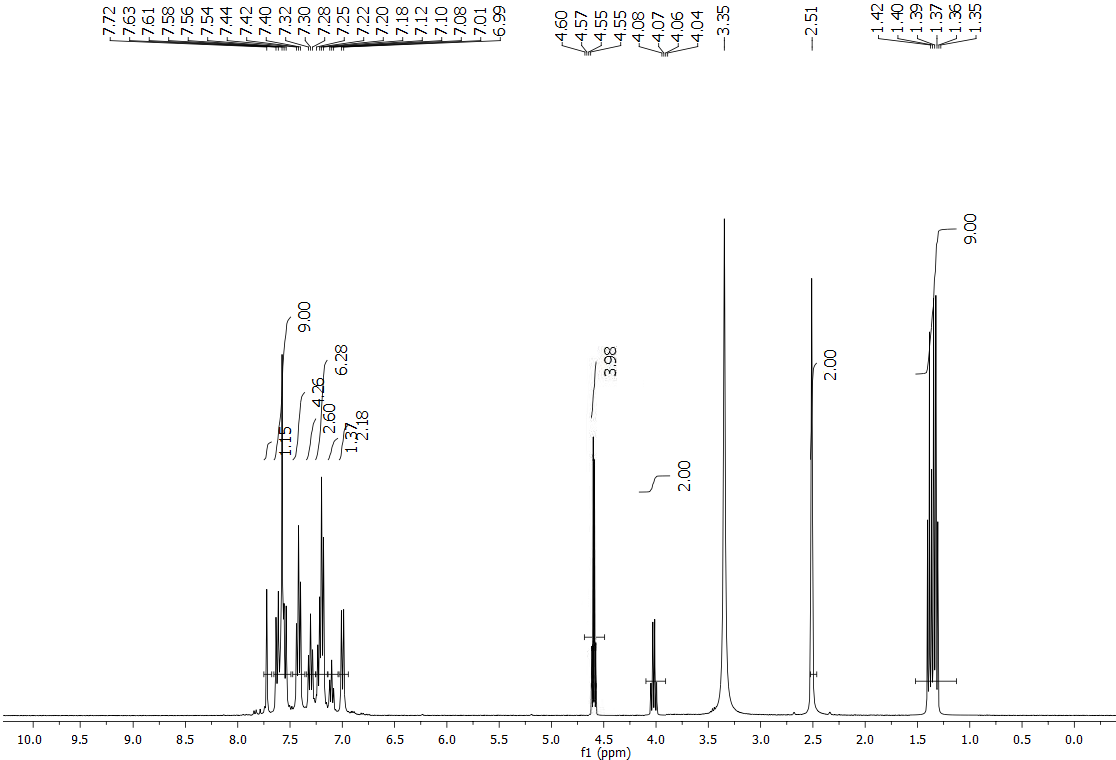


**Figure (S9): ^1^HNMR spectrum of compound 4.**


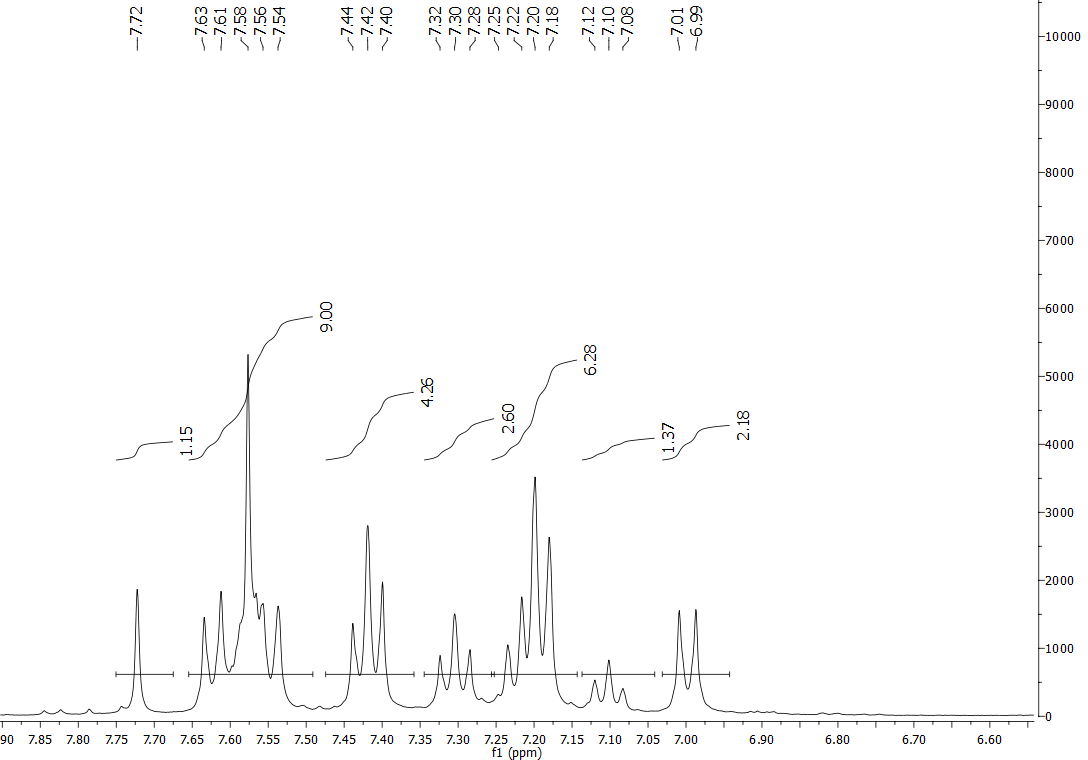


**Figure (S10): ^1^HNMR spectrum of compound 4.**


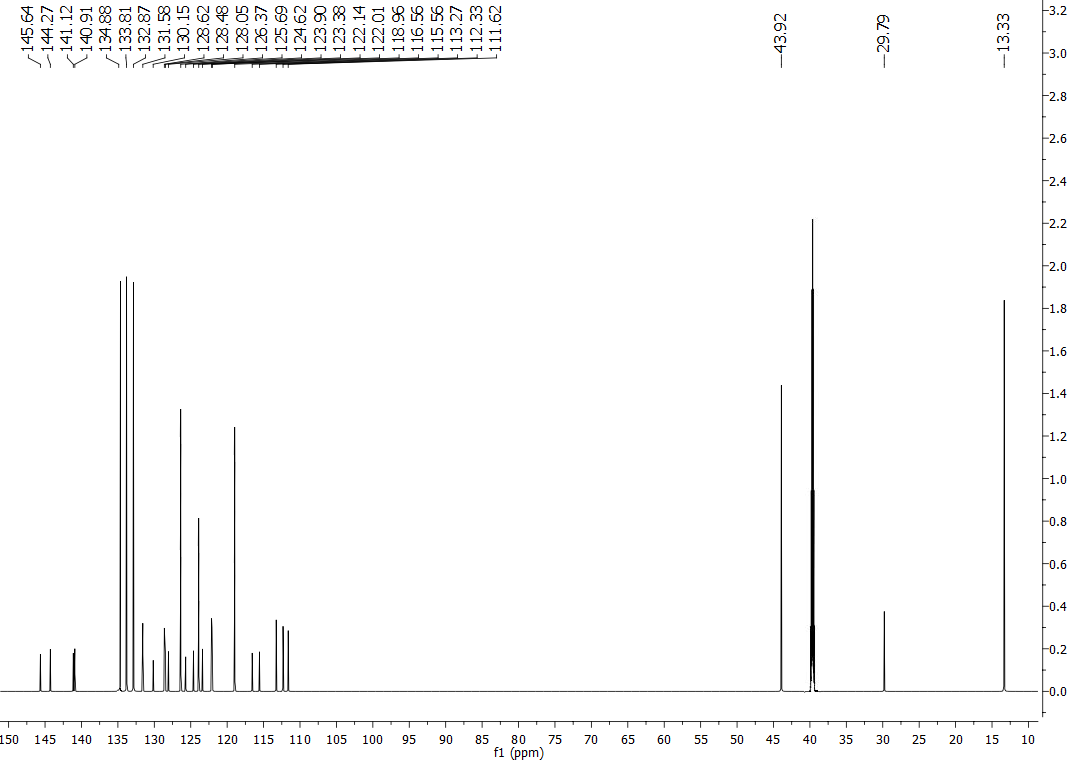


**Figure (S11): ^13^CNMR spectrum of compound 4.**


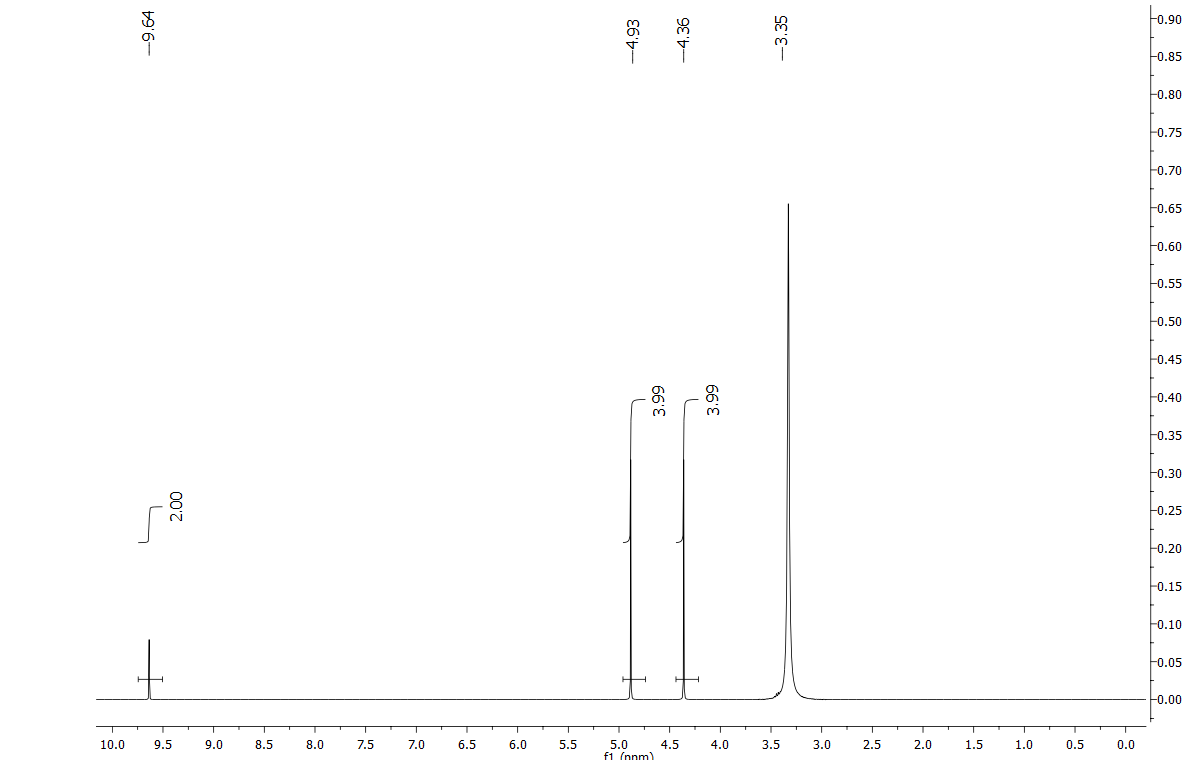


**Figure (S12): ^1^HNMR spectrum of compound 5.**


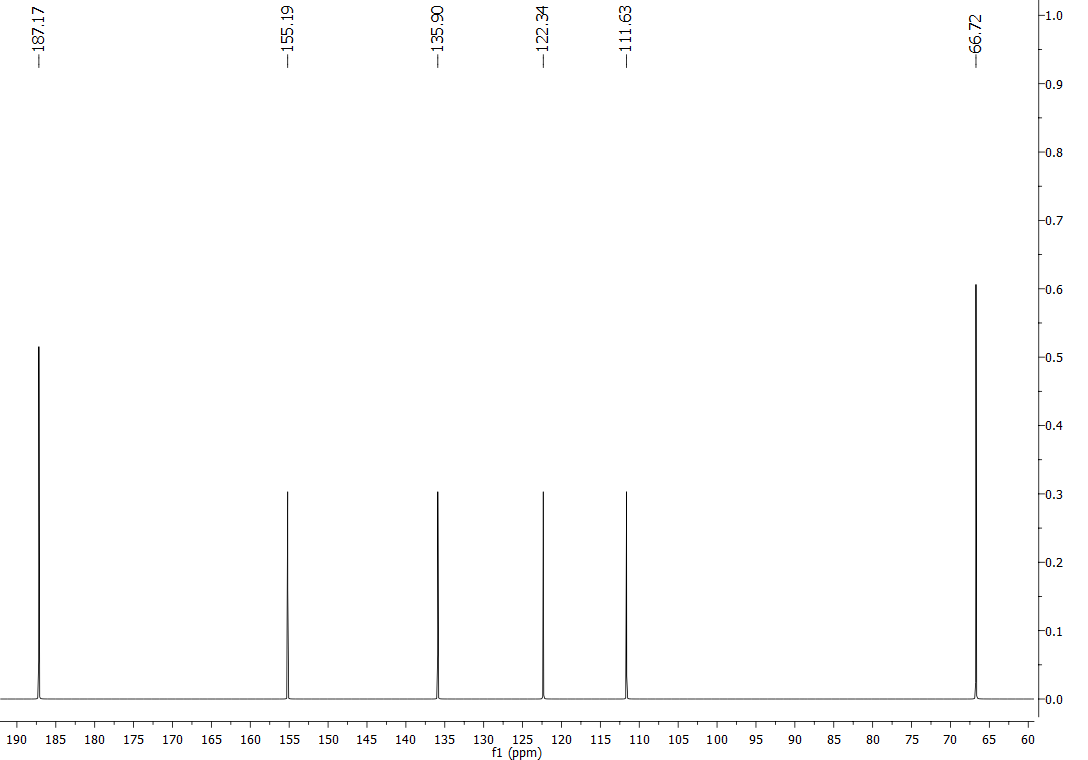


**Figure (S13): ^13^CNMR spectrum of compound** 5.


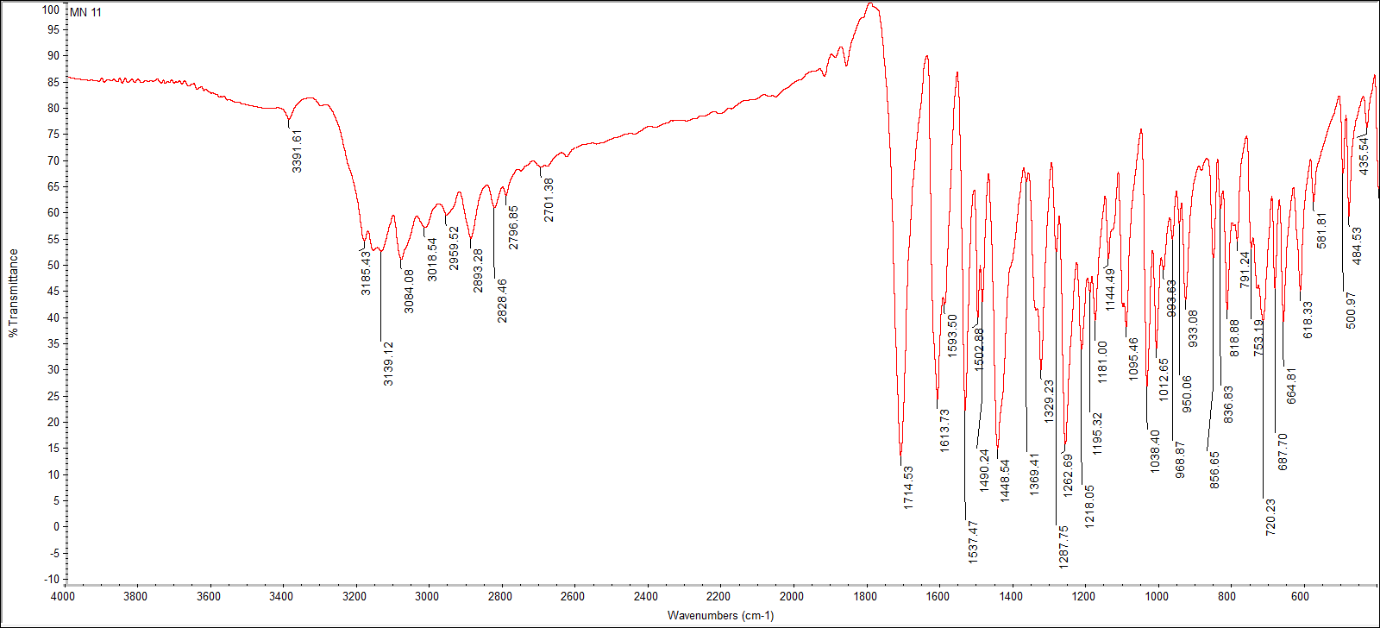

**Figure (S14): IR spectrum of compound 6.**


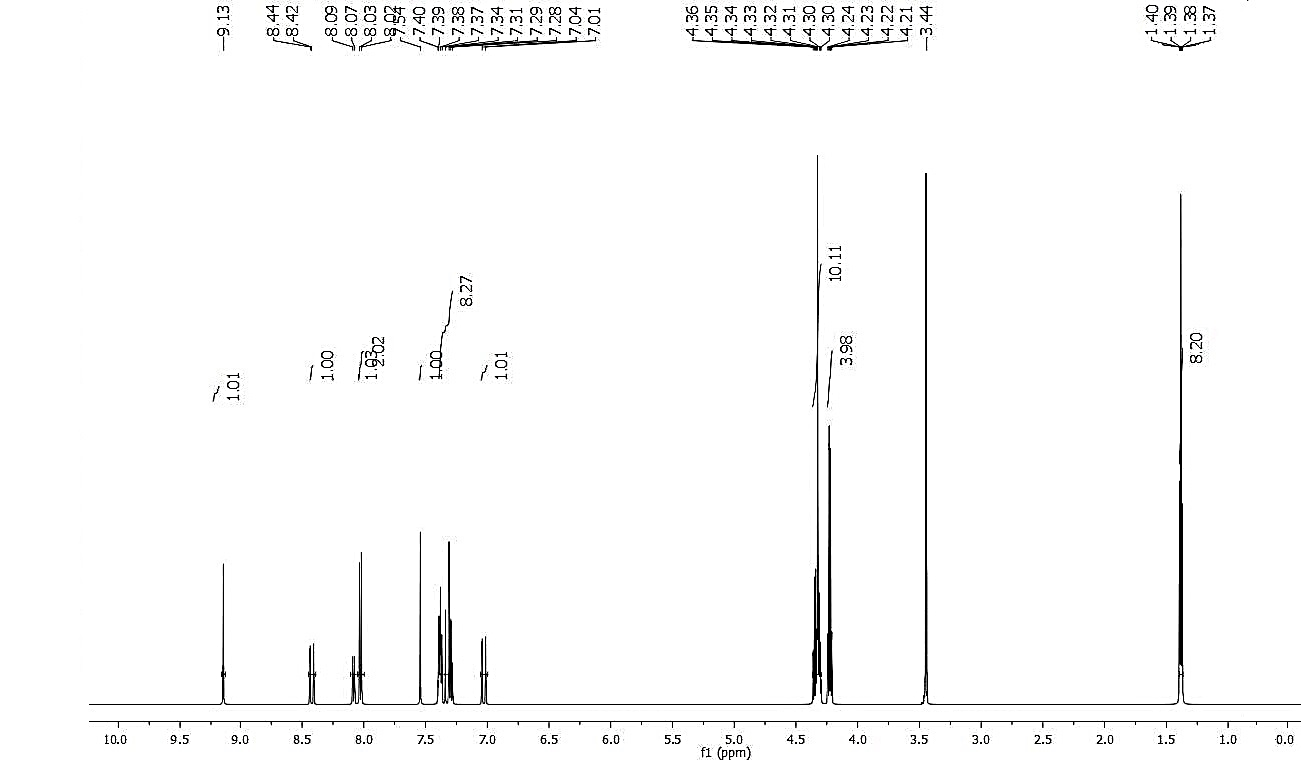

**Figure (S14): ^1^H NMR spectrum of compound 6.**


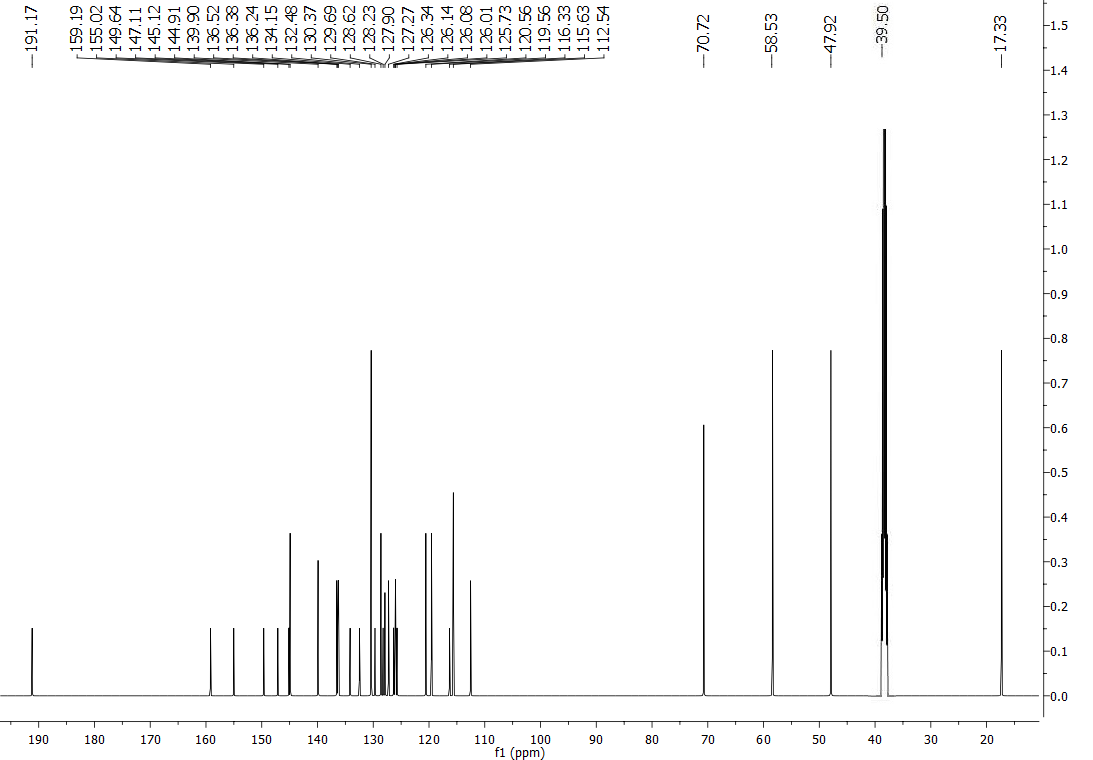

**Figure (S16): C^13^ NMR spectrum of compound 6.**


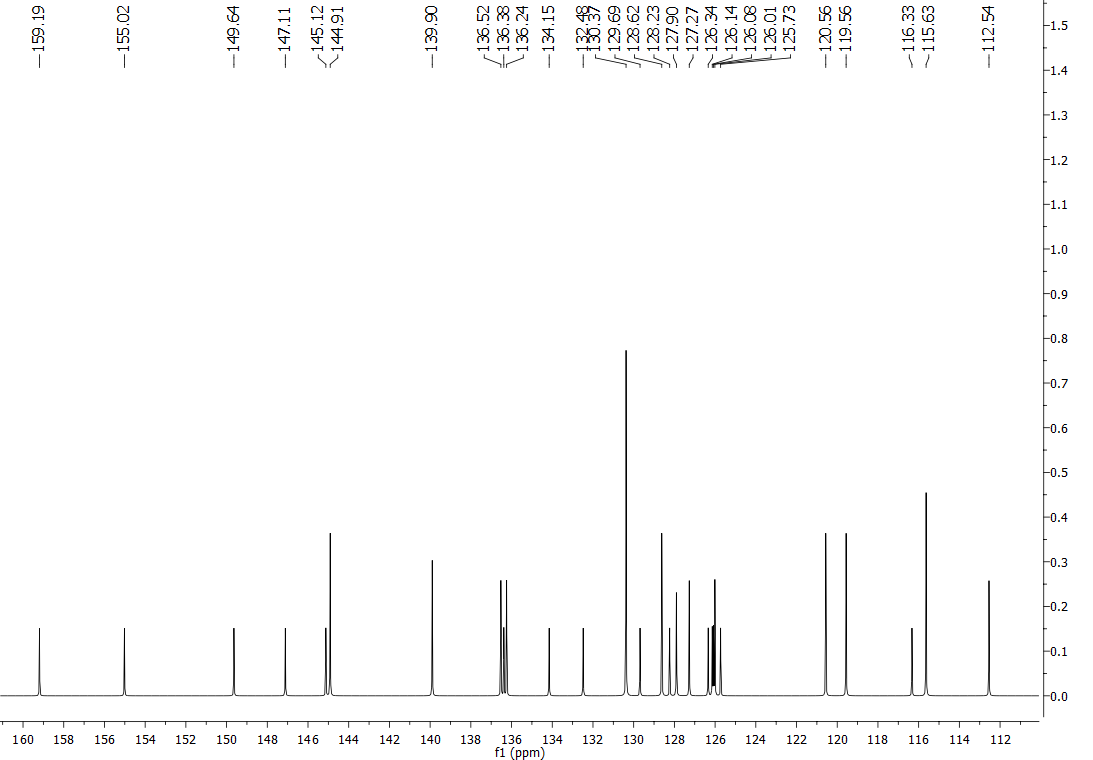

**Figure (S17): Zoom C^13^ NMR spectrum of compound 6.**


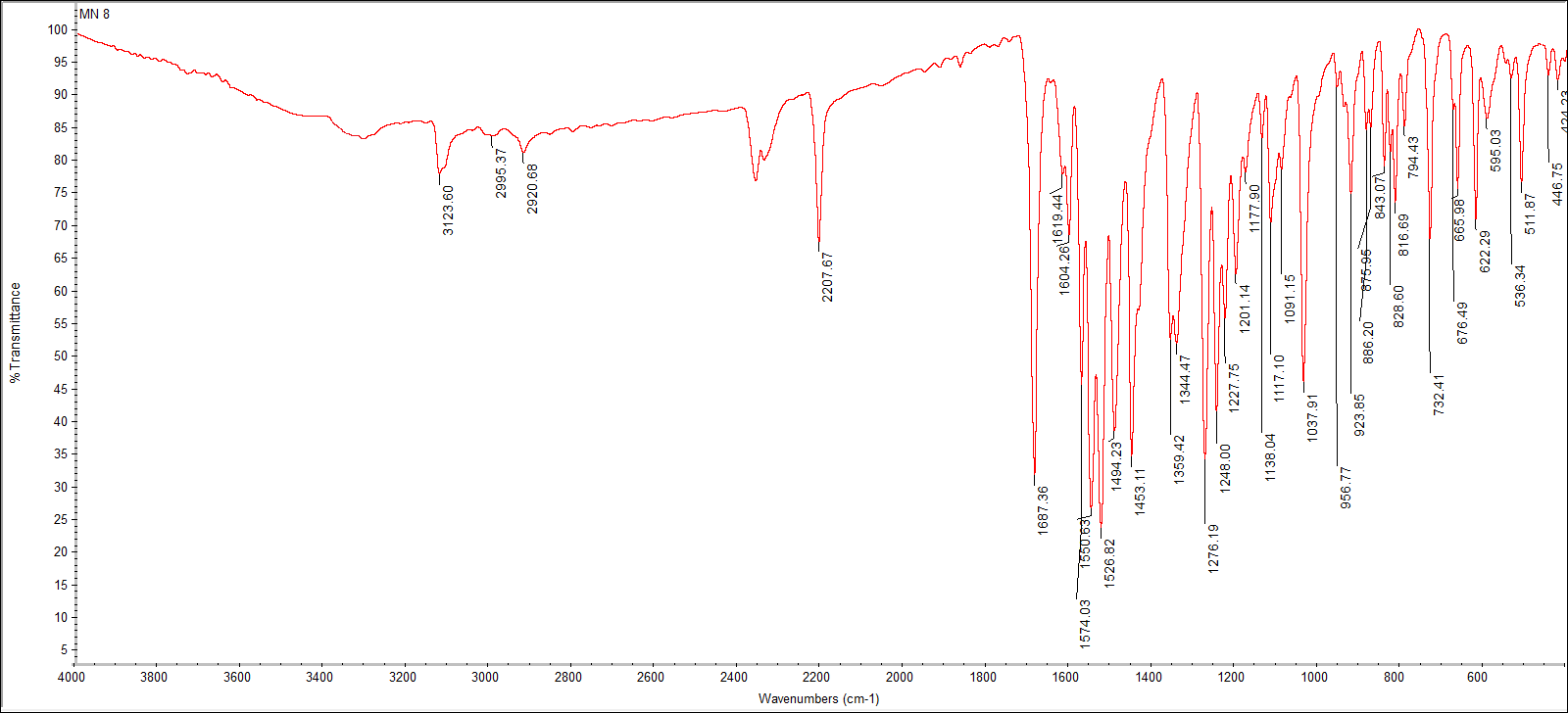

**Figure (S18): IR spectrum of senstizer MS-1.**


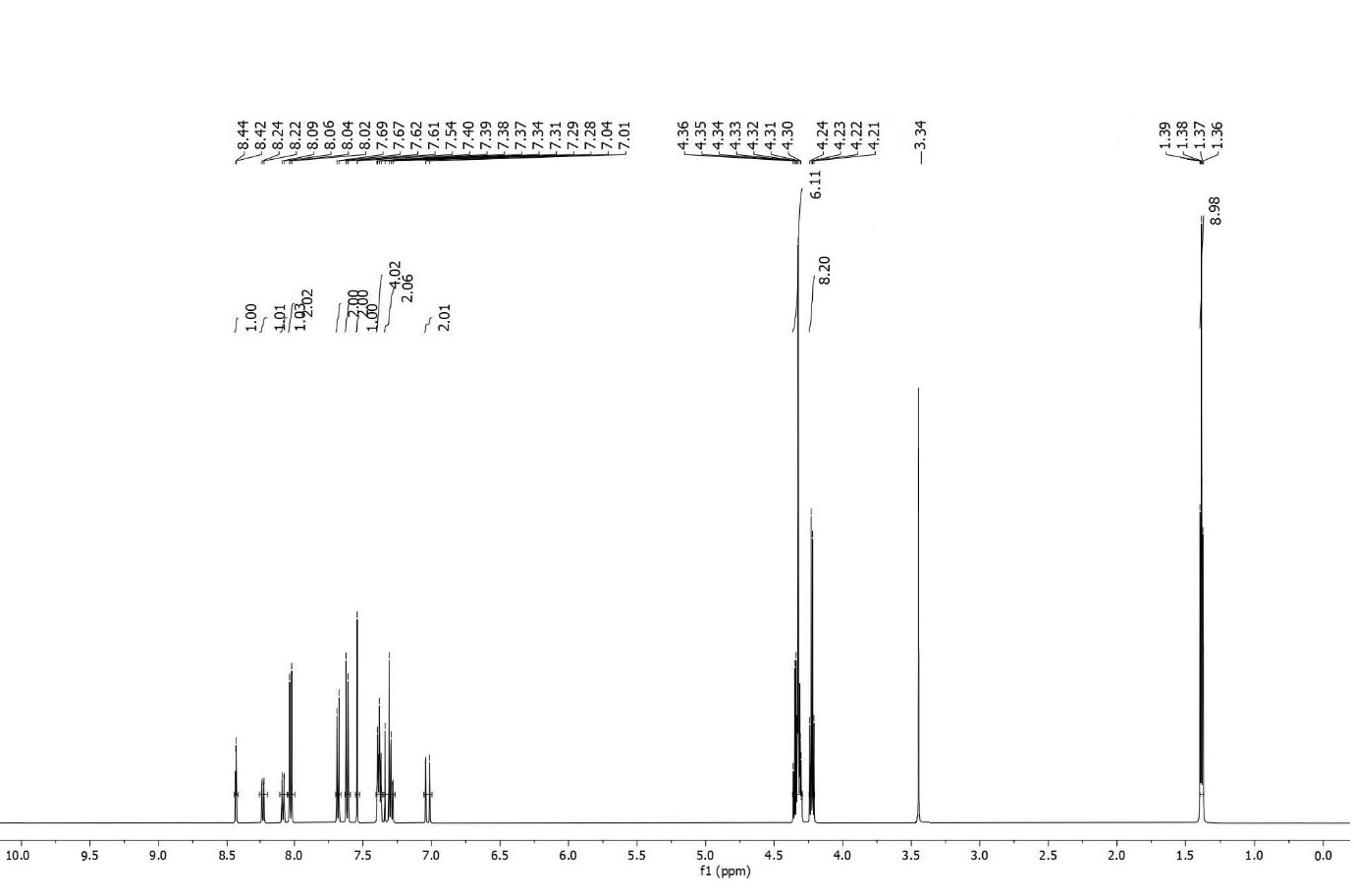

**Figure (S19): ^1^H NMR spectrum of sensitizer MS-1.**


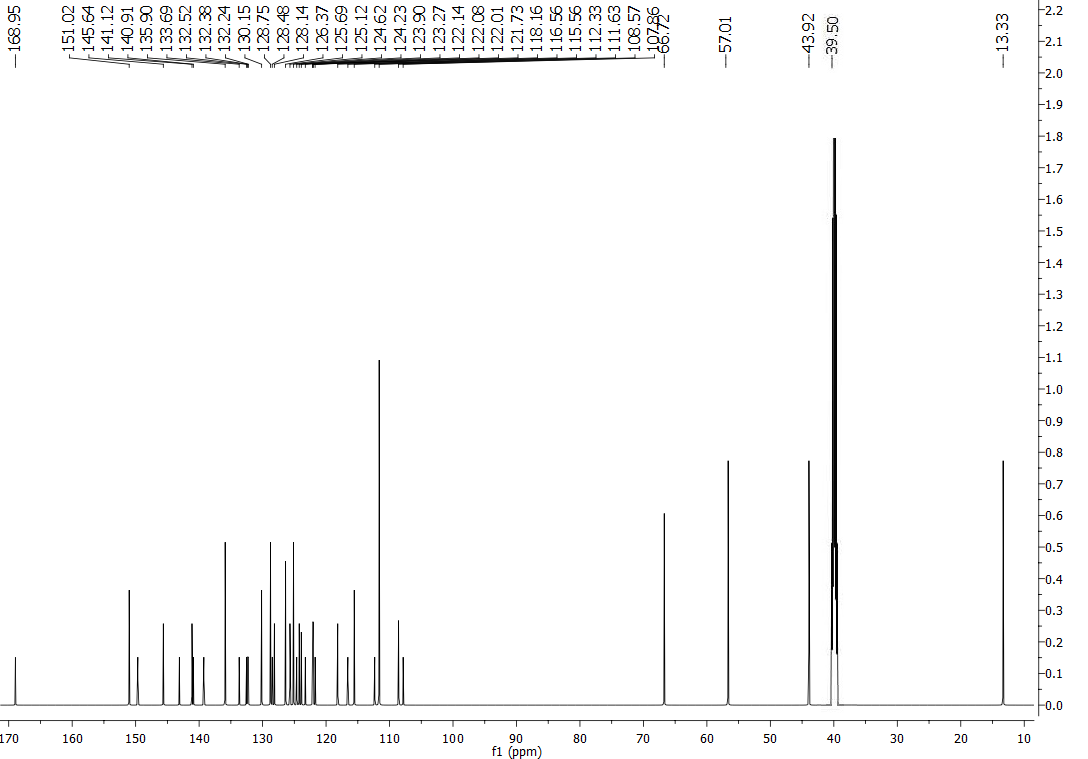

**Figure (S20): ^13^C NMR spectrum of sensitizer MS-1.**


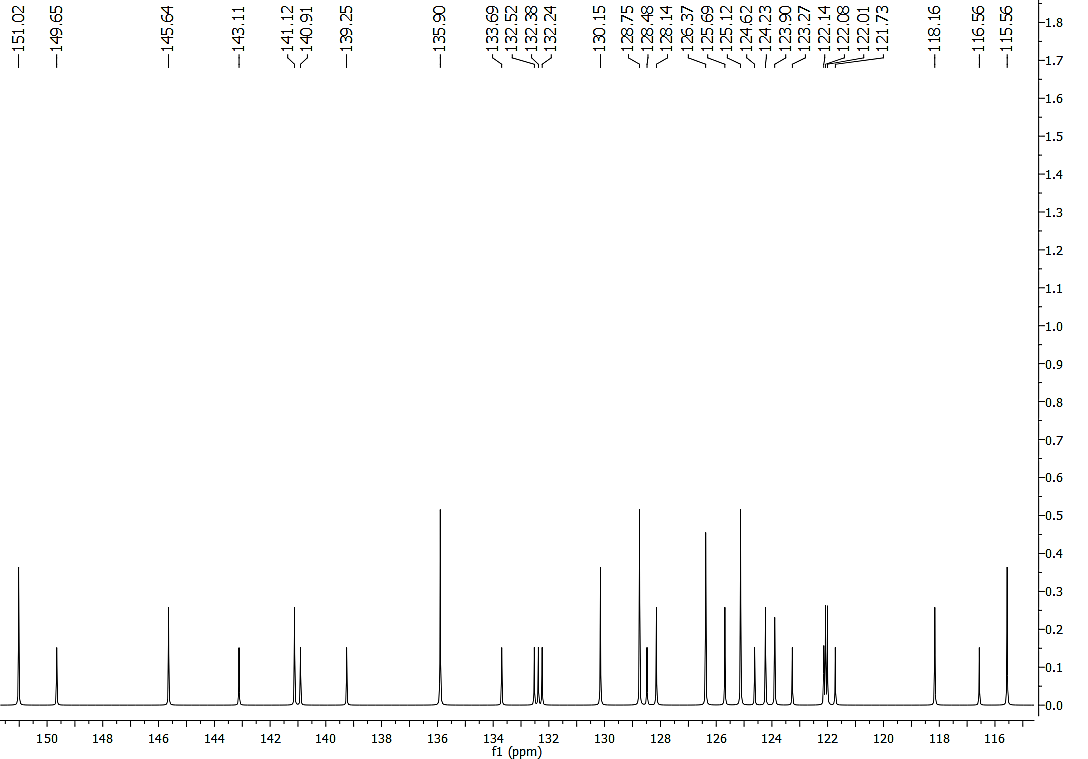

**Figure (S21): Zoom ^13^C NMR spectrum of sensitizer MS-1.**


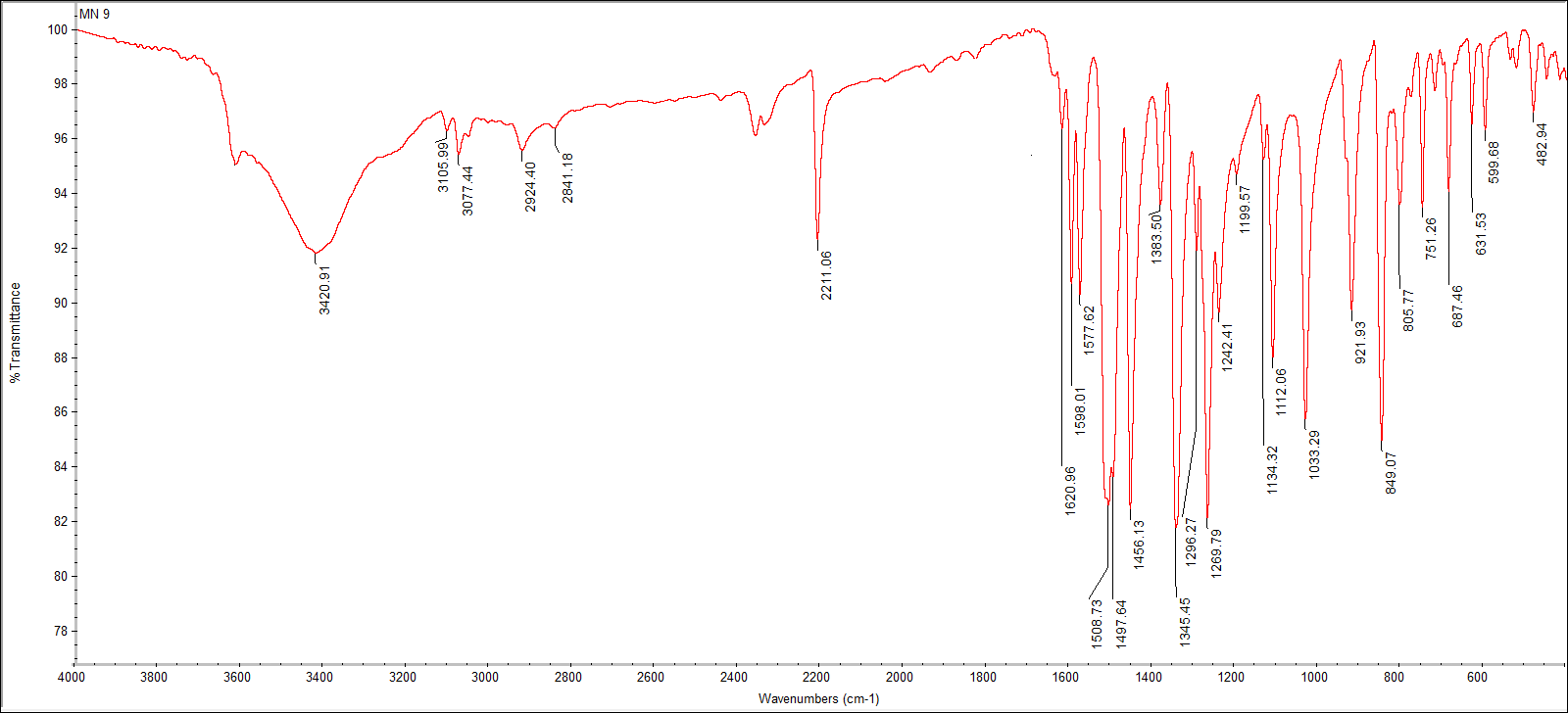

**Figure (S22): IR spectrum of senstizer MS-2.**


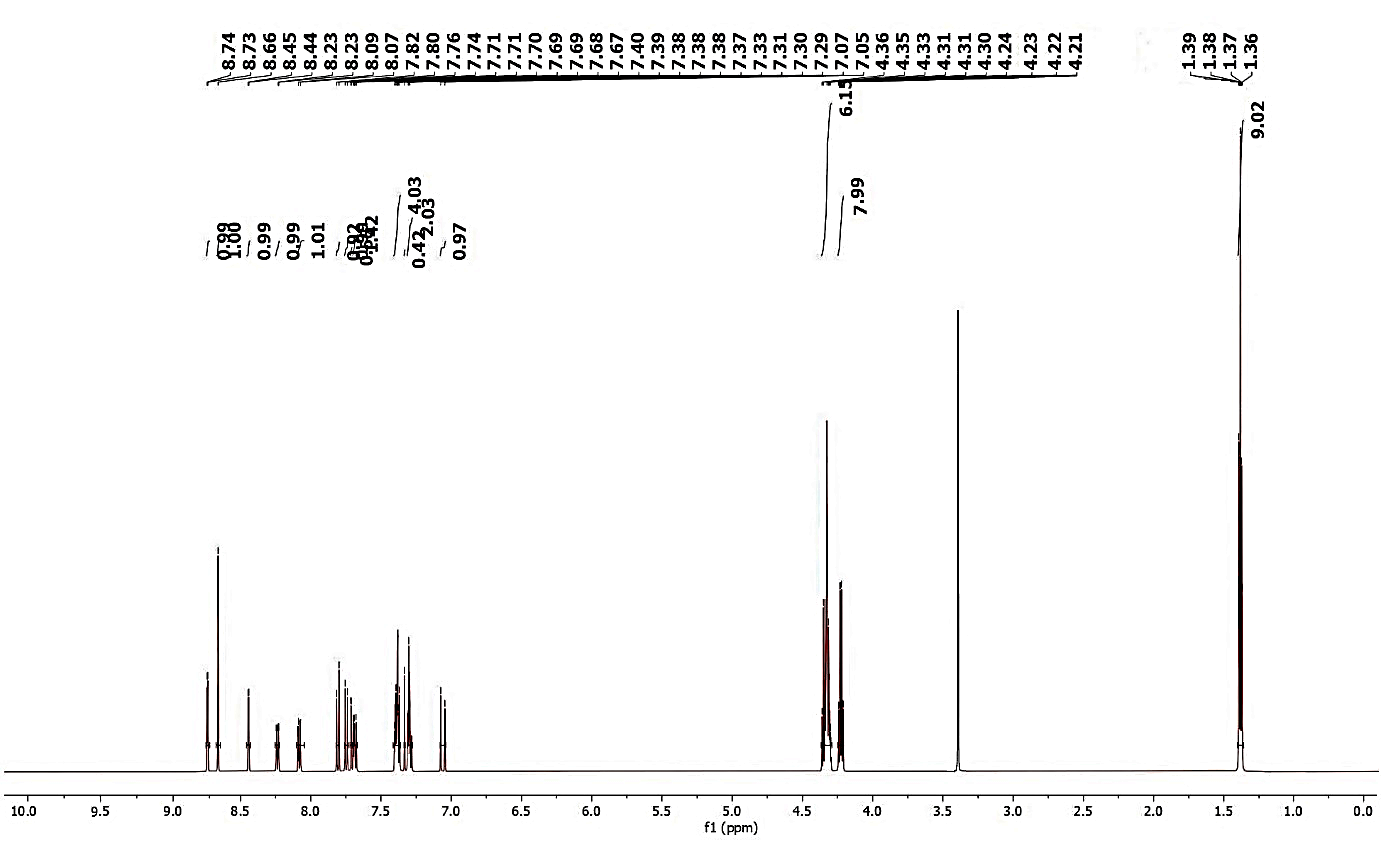

**Figure (S23): ^1^H NMR spectrum of sensitizer MS-2.**


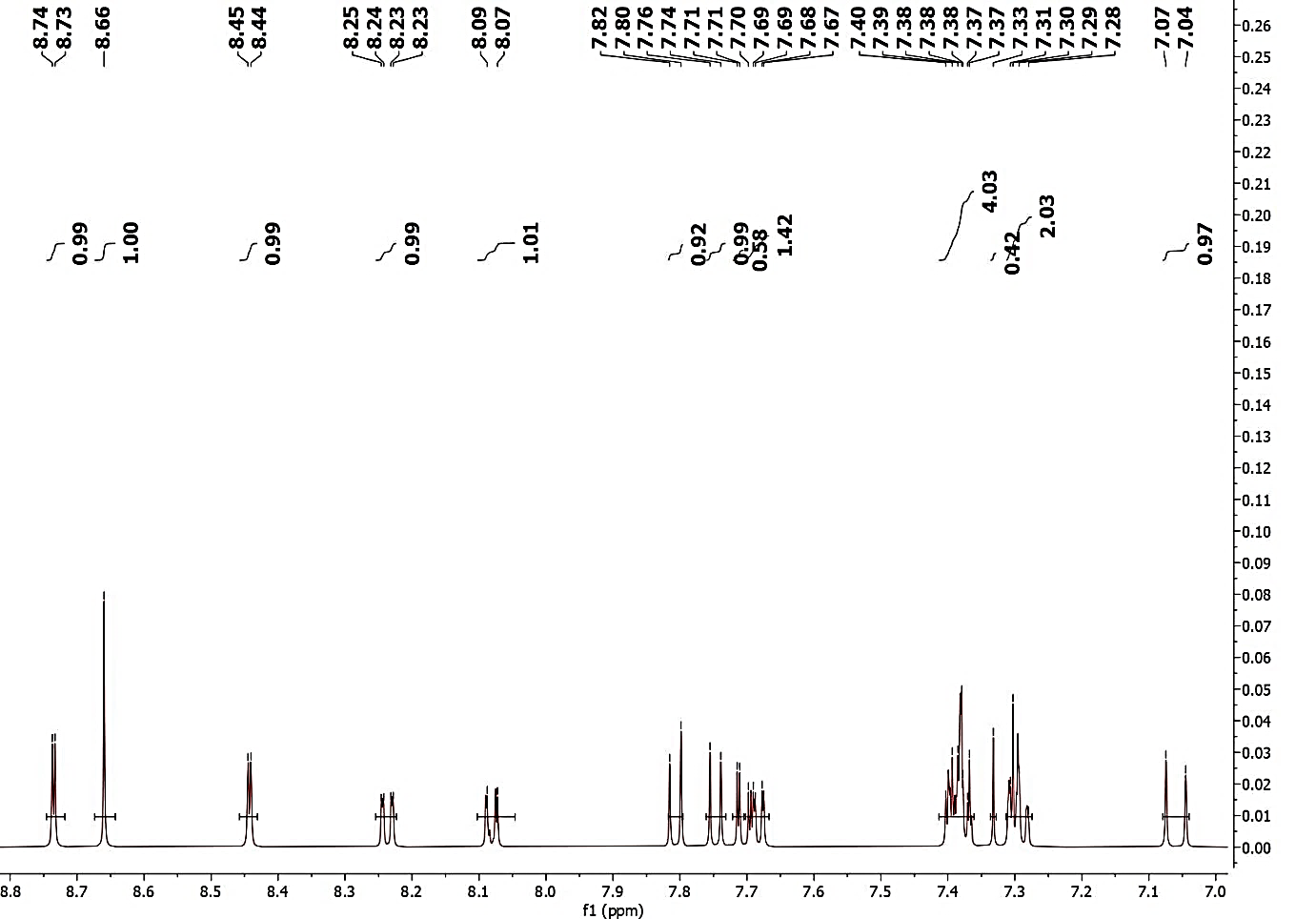


**Figure (S24): Zoom ^1^H NMR spectrum of sensitizer MS-2.**


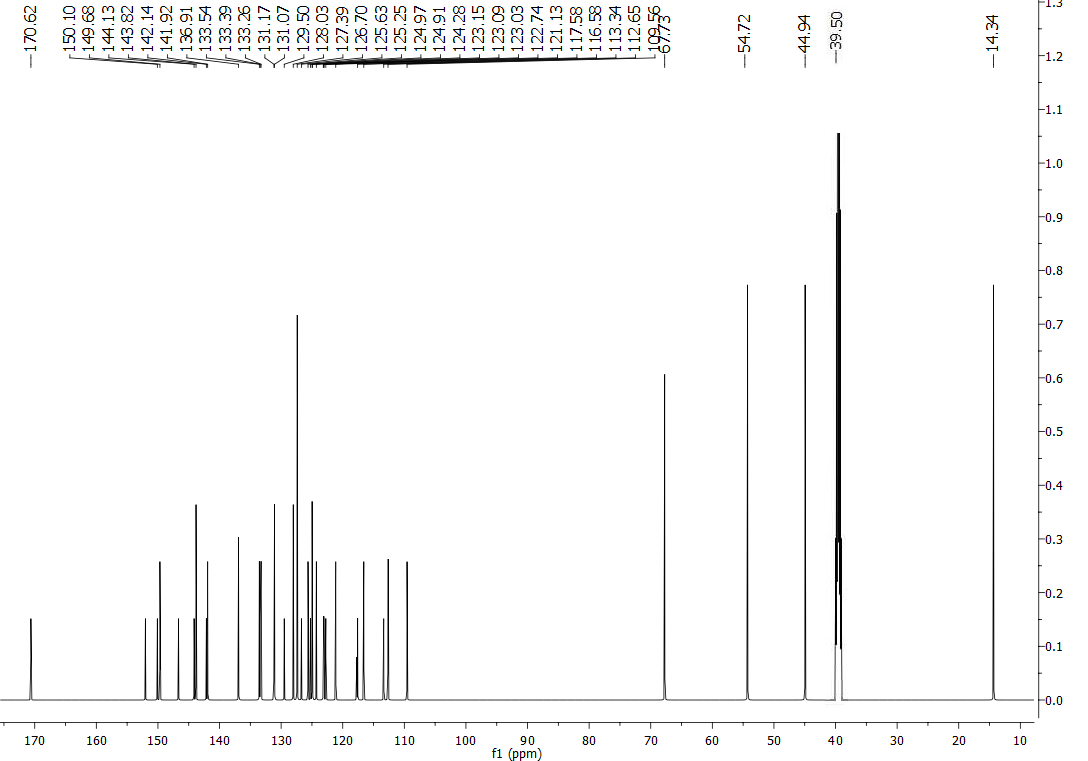

**Figure (S25): ^13^C NMR spectrum of sensitizer MS-2.**


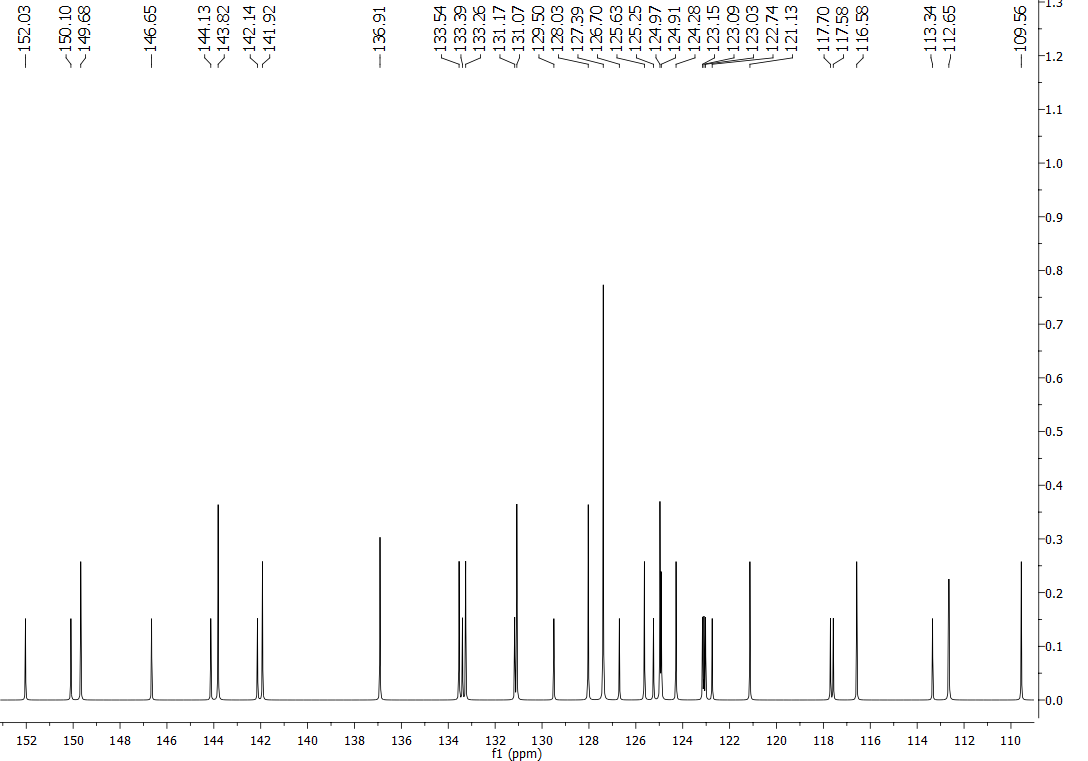

**Figure (S26): Zoom^13^C NMR spectrum of sensitizer MS-2.**

**Figure (S27): Cyclic voltammetry spectra of sensitizers MS-1-2.**

**
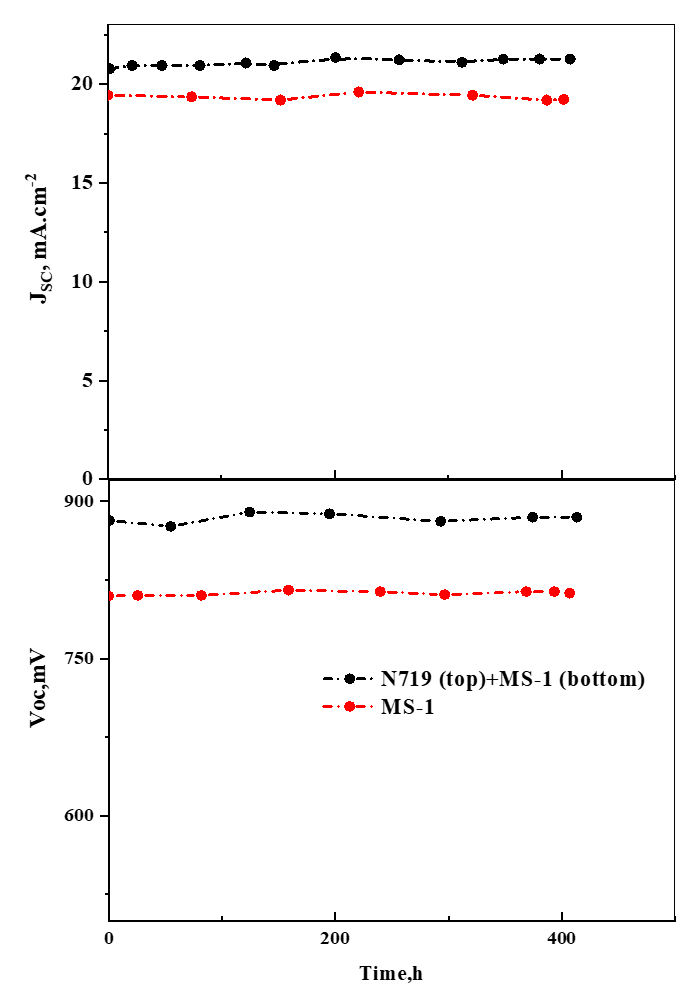
**

**Figure (S28): Jsc vs Voc at certain time interval for MS-1 and N719+MS-1 devices.**

**2. Cell preparations and photovoltaic characterizations**

**2.1. Device assembly and Measurements**

The fabrication of PT-DSSCs involved several intricate steps. First, an electrolyte solution was prepared, containing 0.5 M tert-butylpyridine, 0.05 M I_2_, 0.1 M LiI, 0.1M DMII, 0.5 M BMII, and 0.1M guanidine thiocyanate dissolved in a mixture of acetonitrile and pentanenitrile (85:15, v/v). Separate dye solutions of 0.2 mM **N719** and **MS-1** were prepared using a mixture of chloroform, ethanol, and acetonitrile (3:7:2 v/v). TiO_2_ photoanodes were fabricated by screen-printing 18NR-T and 18NR-AO pastes onto cleaned FTO glass, followed by a programmed temperature calcination process (325°C for 10 min, 375°C for 10 min, 450°C for 25 min, 500°C for 25 min). The cooled films underwent TiCl_4_ treatment (40 mM at 70°C for 30 min) and a final calcination at 450°C for 30 min. These anodes were then sensitized with 0.5 mM solutions of MS-1 or N719 for 16 hours. Counter electrodes were prepared by spin-coating a 0.02 M chloroplatinic acid solution in isopropyl alcohol onto FTO glass and calcining at 600°C for 25 min. The T-DSSC devices were assembled by connecting top and bottom cells in parallel using double-sided Pt counter electrodes, with MS-1 and N719 serving as the photoanode sensitizers for the top and bottom cells, respectively. []

Photovoltaic and incident photon-to-current efficiency (IPCE) measurements were made on sandwich cells, which were prepared using TiO_2_ coated working electrodes and platinum coated counter electrodes and were sealed using a 40 μm Syrlyn spacer through heating of the polymer frame. The redox electrolyte (Solaronix, Iodolyte HI-30) consisted of a solution of 0.6 M DMPII, 0.05 M I_2_, 0.1 M LiI and 0.5 M TBP in acetonitrile.

2.3. Photovoltaic measurements

Photovoltaic measurements of sealed cells were made by illuminating the cell through the conducting glass from the anode side with a solar simulator (WXS-155S-10) at AM 1.5 illuminations (light intensity: 100 mW cm^−2^).

2.4. Incident photon to current efﬁciency (IPCE) conversion

IPCE measurements were made on a CEP-2000 system (Bunkoh-Keiki Co. Ltd.). IPCE at each wavelength was calculated using Equation 1, where *I_SC_* is the short-circuit photocurrent density (mA. cm^−2^) under monochromatic irradiation, q is the elementary charge, λ is the wavelength of incident radiation in nm and P0 is the incident radiative flux in W/m^2^.

$$\mathrm{IPCE}\left( \lambda\right)=1240\left( \frac{I_{\mathrm{SC}}}{q\lambda P_{o}} \right) (\mathbf{1})$$

2.5. Cyclic voltammetry

The cyclic voltammetry (CV) measurements were performed using a computer-controlled CHI660C electrochemical workstation with a three-electrode system (The redox potentials of dyes were measured at the same concentration of electrolyte (0.1 M tetra-n-butylammonium hexafluorophosphate (TBAPF6)) in anhydrous acetonitrile (oxidation) or tetrahydrofuran (reduction), Reference electrode: Ag/AgCl; Internal reference: calibrated with ferrocene/ferrocenium (Fc / Fc^+^); Counter electrode: Pt).

**3. Molecular Modeling**

Equilibrium molecular geometries of **MS-1-2** calculated using the Becke's three parameter hybrid functional, Lee–Yang–Parr's gradient corrected correlation functional (B3LYP) and (6-311g(d, p)) [1, 2, 3, 4]. The geometry optimization calculations were followed by energy calculations using time-dependent density functional theory (TD-DFT) utilizing the energy, functional B3lyp and the basis set 6-311g (d, p). The solvent (DMF) effect was accounted for by using the conductor-like polarizable continuum model (C-PCM), implemented in Gaussian 09. ت

**References**

[1] G. Melikian, F. Rouessac, C. Alexandre, Synth Commun. 1993, 23, 2631

[2] A. D. Becke, Phys. Rev. A **1988**, 38, 3098.

[3]C. T. Lee, W.T. Yang, R.G. Parr, Phys. Rev. B. **1988**, 37, 785

[4] N. Godbout, D.R. Salahub, J. Andzelm, E. Wimmer, Optimization of Gaussian-type basis-sets for local spin-density functional calculations .1. Boron through neon, optimization technique and validation. Can. J. Chem.-Rev. Can. Chim. **1992**, 70, 560-571.
